# Acute Effect of Night Shift Work on Endothelial Function with and without Naps: A Scoping Review

**DOI:** 10.3390/ijerph20196864

**Published:** 2023-09-29

**Authors:** Paul D. Patterson, Jacob C. Friedman, Samuel Ding, Rebekah S. Miller, Christian Martin-Gill, David Hostler, Thomas E. Platt

**Affiliations:** 1 Department of Emergency Medicine, School of Medicine, University of Pittsburgh, Pittsburgh, PA 15261, USA; 2 Department of Community Health Services and Rehabilitation Sciences, School of Health and Rehabilitation Sciences, University of Pittsburgh, Pittsburgh, PA 15261, USA; 3 Health Sciences Library System, University of Pittsburgh, Pittsburgh, PA 15261, USA; 4 Department of Exercise and Nutrition Sciences, School of Public Health and Health Professions, University at Buffalo, The State University of New York, Buffalo, NY 14214, USA; dhostler@buffalo.edu

**Keywords:** shift work, endothelial function, cardiovascular disease, napping, scoping literature review

## Abstract

We examined the breadth and depth of the current evidence investigating napping/sleeping during night shift work and its impact on non-invasive measures of endothelial function. We used a scoping review study design and searched five databases: Ovid Medline, EMBASE, Ovid APA PsycInfo, Web of Science Core Collection, and EBSCO CINAHL. We limited our search to English language and publications from January 1980 to September 2022. Our reporting adhered to the PRISMA-ScR guidance for scoping reviews. Our search strategy yielded 1949 records (titles and abstracts) after deduplication, of which 36 were retained for full-text review. Five articles were retained, describing three observational and two experimental research studies with a total sample of 110 individuals, which examined the non-invasive indicators of endothelial function in relation to the exposure to night shift work. While there is some evidence of an effect of night shift work on the non-invasive indicators of endothelial function, this evidence is incomplete, limited to a small samples of shift workers, and is mostly restricted to one measurement technique for assessing endothelial function with diverse protocols. In addition, there is no identifiable research investigating the potential benefits of napping during night shift work on non-invasive measures of endothelial function.

## 1. Introduction

Cardiovascular disease (CVD) is the leading cause of death in the U.S. and globally [1]. The risk of CVD and the incidence of hypertension over a 5- to 10-year period is higher among shift workers compared to traditional daylight workers [2,3]. A shift work schedule, compared to a non-shift work daytime schedule, is associated with a 23% increased risk of myocardial infarction (risk ratio 1.23, 95%CI 1.15, 1.31) and a 5% increased risk of ischemic stroke (risk ratio 1.05, 95%CI 1.01, 1.09) [4,5]. The risk of myocardial infarction, coronary-related mortality, or hospital admission due to coronary artery disease is greatest among night shift workers compared to other shift schedules (risk ratio 1.41, 95%CI 1.13, 1.76) [5]. Organizations such as the U.S. National Institutes for Occupational Safety and Health (NIOSH), the U.S. Federal Emergency Management Agency, the World Health Organization, and the International Labour Organization (ILO) support the risk reduction in CVD among shift workers [6,7,8]. Common risk mitigation strategies include the frequent assessment of non-invasive indicators of CVD, such as blood pressure (BP) [9]. The monitoring of other pre-clinical indicators, such as endothelial function, may provide greater insight into CVD risk and disease progression among shift workers [10,11,12,13], yet little is known about the evidence for the indicators of endothelial function, including sensitivity to on-shift interventions such as napping.

The endothelium is a single-layer of cells that line the vascular system, including arteries, veins, and capillaries, and it is an important modulator of vascular tone, BP, and other critical functions that impact cardiovascular homeostasis [14]. The endothelium plays an important role in coagulation and clotting processes, which has an impact on the development of atherosclerosis [15]. Endothelial dysfunction can manifest as changes in molecule secretion, inflammation in the vessel wall, and as increased BP [11]. Prior studies report circadian variation in endothelial function marked by the attenuation of function in the early morning compared to evening [16]. Long-term indicators of endothelial dysfunction include arterial stiffness and evidence of atherosclerosis [15].

Advantages of assessing endothelial function, especially non-invasively, include (1) less risk compared to invasive techniques like the intra-coronary infusion of acetylcholine to measure arterial diameter [17]; (2) mostly all CVD risk factors are linked to endothelial dysfunction [15,18]; (3) because endothelial dysfunction is systemic, dysfunction detected in the peripheral vasculature correlates with dysfunction in the coronary arteries [19]; (4) numerous studies using non-invasive techniques report statistically significant associations with CVD outcomes [18,20,21]; and (5) the best practice for monitoring other indicators, like BP, often require prolonged 24 h monitoring for diagnostic purposes [22], which may not be feasible in all workplace wellness and shift work settings.

Shift work is described as working hours outside of what may be defined as a normal working day (e.g., 9 a.m. to 5 p.m.) [23]. Night shift work encompasses work beyond midnight for 3 or more hours [23]. According to one estimate, 16.8% of all full-time wage and salary workers in the United States (U.S.) are shift workers [24]. Other estimates suggest that one-fifth of workers in the U.S. and European Union (E.U.) are shift workers, with as much as one-third of employees in the U.S. and E.U. working non-standard hours and/or >48 h per week [25]. Shift work schedules such as night shifts, long duration shifts, and rotating shift work are linked to shortened sleep (by 1–2 h compared to non-shift workers), disrupted sleep, and poor sleep quality [23]. Sleep loss associated with shift work, especially night shift work, has been shown to disrupt normal patterns in BP and affect the normal functions of the endothelium [13,26,27]. The repeated disruptions of BP and endothelial function associated with the frequent exposure to shift work may exacerbate the risk of CVD [10,28].

The studies of acute (e.g., days) and longer term (e.g., years) exposure to shift work show an association between shift work and endothelial dysfunction [10,13,28,29]. Suessenbacher and colleagues reported reduced peripheral arterial tone—a non-invasive measure of endothelial function—among shift workers with a mean of 9.9 years (SD5.3) of work experience when compared to non-shift workers with a mean of 14.5 years (SD8.9) of work experience [10]. A separate study of nurses showed a significant decrease in endothelial function after three consecutive night shifts when compared to pre-night shift work (baseline) [29]. In this study, a longer history of night shift work was strongly associated with a decrease in endothelial function, as measured via flow-mediated dilation (FMD) [29]. Charles and colleagues compared measures of FMD assessed 7 years apart in police officers and determined that officers who worked night shifts experienced greater declines in endothelial function than officers who worked daylight or afternoon shifts [13].

Napping during night shifts briefly restores normal (wake/sleep) patterns in BP [27,30], which may directly or indirectly impact the relationship between sleep loss during night shift work and endothelial function [31]. A normal pattern of BP is marked by a 10–20% decrease (dip) in BP during sleep/nighttime hours relative to the wake/daytime hours [32]. Napping refers to “*sleep periods at least 50% shorter than an individual’s average nocturnal sleep length*” [33,34]. A study of 56 Emergency Medical Services (EMS) night shift workers showed that a nap of 60 min or longer during night shift work was associated with the restoration of normal BP dipping, for most, during the night shift work hours [27]. A recent meta-analysis of 24 h shifts showed that Systolic BP (SBP) and Diastolic BP (DBP) were significantly higher during wakefulness on the long duration shift compared to sleep during this shift [30]. This meta-analysis showed that among those who slept during the 24 h shift, the pooled mean dip in SBP was 14.8% (95%CI 11.4, 18.2) and the mean dip in DBP was 17.1% (95%CI 13.6, 20.6) [30]. Evidence of a napping effect on endothelial function could impact how and when it is assessed in relation to the exposure to shift work.

We sought to examine the breadth and depth of the current evidence investigating napping/sleeping during night shift work and its impact on non-invasive measures of endothelial function. Our findings will inform others guided by the work of the ILO, NIOSH, and other organizations (public and private) who regularly monitor and investigate the working conditions and/or offer guidance or resources for employers responsible for mitigating threats to shift worker health and safety [25,35,36]. Our findings will inform researchers who address NIOSH’s Strategic Goals, Intermediate Goals, and Activity Goals for occupational health and safety, specifically Intermediate Goals 1.10, 1.11, 1.13, and 7.17, and 7.1–7.12, 7.14, 7.5, 7.6, and 7.10 of the NIOSH Strategic Plan for 2019–2026, and also the corresponding Activity Goals, which target work arrangement and scheduling as risk factors for illnesses like CVD [36]. The findings from our review will inform researchers as well as decision-makers who seek to address the ILO’s policy suggestions, such as “Developing balanced working time arrangements” that promote health and safety [25]. In addition, our review will inform those following or taking action on NIOSH’s Future of Work Initiative, which promotes collaboration and new research to address workplace-related risks created by job arrangements and organizational design [35]. The Initiative also promotes creating risk profiles and new approaches to mitigate risk with “new solutions and practical approaches” [35]. Irrespective of the goal, objective, initiative, or policy suggestion selected, researchers and individuals responsible for policy-relevant decisions should be cognizant of the best available evidence (“be aware of what is out there”) for measuring and quantifying the indicators of risks (or for assessing intervention impact) with respect to a key pre-clinical indicator of CVD. In this review, we will identify and characterize the breadth and depth of evidence related to a potentially useful and clinically meaningful approach to assessing risk (or intervention impact) for CVD.

## 2. Materials and Methods

### 2.1. Study Design

Our review was initially guided by a single research question framed in the traditional Population, Intervention/Exposure, Comparison, Outcome (P.I.C.O.) format: “*Does napping/sleeping during night shift work mitigate the impact of sleep deprivation/sleep restriction on indicators of endothelial function*?” Following a systematic search of the literature identifying no studies directly answering this question, we reframed our search as a scoping review of the literature aimed at investigating the breadth and depth of the current evidence evaluating the relationship between napping/sleeping during night shift work and endothelial function. We used a scoping review study design given its utility for investigations focused on the depth and breadth of the evidence, the need to map gaps in the evidence, and because of the uncertainty regarding the potential number of original research studies that addressed our P.I.C.O. [37].

### 2.2. Search Strategy

As prescribed [37], we conducted a comprehensive search of multiple databases (*n* = 5) for original research relevant to our original P.I.C.O. research question: Ovid Medline, EMBASE, Ovid APA PsycInfo, Web of Science Core Collection, and EBSCO CINAHL. For each database search, we used controlled vocabulary and keywords for the concepts of endothelial function and shift work, and we limited our search date range from 1 January 1980 to 8 September 2022. The search results were deduplicated using the Amsterdam Efficient Deduplication method [38] and then uploaded into Endnote citation management software. The records were then deduplicated a second time using the Bramer method [39]. See Appendix A for details of our search strategy stratified by database. The records resulting from deduplication were then uploaded to DistillerSR (DistillerSR Inc., Ottawa, ON, Canada).

### 2.3. Screening Methodology

The adjudication of records was guided by a priori inclusion/exclusion criteria applied by two independent record reviewers. Agreement between reviewers during the record screening was assessed with the Kappa statistic. As prescribed [37], the records retained during the title/abstract screening were examined in full-text form, adjudicated for inclusion or exclusion, and the data from the included full-text articles were extracted into tables and verified by two investigators (See Appendix B). The extracted data were descriptively synthesized, where the reasons for excluding articles appear in Appendix C, and the presentation of results adhered to the PRISMA-ScR flow diagram (See Appendix D) [40]. We also performed bibliography searches during the review of full-text articles to identify potentially relevant research not identified during the record screening. Our protocol was not published or registered in advance.

### 2.4. Population of Interest

Our population of interest was shift workers from any occupation (e.g., public safety, healthcare, transportation, manufacturing, or related shift worker groups). Original research studies that involved military personnel as study participants were included.

### 2.5. Intervention/Exposure of Interest

The intervention or exposure of interest was napping during night shift work, long duration shifts (e.g., 24 h), or during simulated night shifts or long duration shift work.

### 2.6. Comparison of Interest

The comparison of interest was on the impact of napping/sleeping versus no napping/sleeping during night shift work on endothelial function.

### 2.7. Outcomes of Interest

Our primary outcome of interest was the indicators of endothelial function or dysfunction measured with non-invasive techniques. Non-invasive assessments include brachial arterial reactivity tests that involve ultrasound, venous occlusive plethysmography that involve BP cuffs placed on the lower and/or upper extremities, and peripheral tonometry, which involves placing probes on a subject’s fingers to assess microvasculature blood flow before, during, and after occlusion of the upper extremity [14,41]. The gold standard measurement of endothelial function involves the invasive assessment of arterial diameter, blood flow, and vascular resistance with intra-coronary infusion of acetylcholine [17]. Biomarkers such as IL-6, C-reactive protein (CRP), and syndecan-1, among others, are indicators of endothelial activation or dysregulation [42,43]. We focused on non-invasive assessments of endothelial function given greater feasibility, compared to invasive methods, of replicating the use of non-invasive devices in future research with diverse shift worker populations.

### 2.8. Analysis

As prescribed [37,40], we used a narrative and descriptive approach to analyze the retained literature. We approached the analyses of the retained literature in this way given that previous research reports that two-thirds of the literature screened for studies that involve systematic reviews and other types of reviews often do not report on the comparison of interest [44]. In this scoping review, our comparison of interest focused on the impact of napping/sleeping during night shifts versus no napping/sleeping and the effect on endothelial function. While we anticipated that a few of the articles reviewed would address all aspects of our P.I.C.O., we took particular interest in the articles (studies) that met most of our P.I.C.O. elements. We closely examined the methods of these articles and reported the findings for the purposes of highlighting important gaps in the evidence. We refer to the articles (studies) that met most of the elements of our P.I.C.O., but not all, as ‘ancillary.’ Highlighting such gaps may offer much needed guidance for future studies. In this analysis, and as recommended by others [37,40], we charted and outlined key findings of all the retained articles in evidence tables (see Appendix B), and below, in the Results and Discussion sections, we use a narrative format to describe important methodological gaps in these studies for the benefit of future research.

## 3. Results

**Figure 1:**
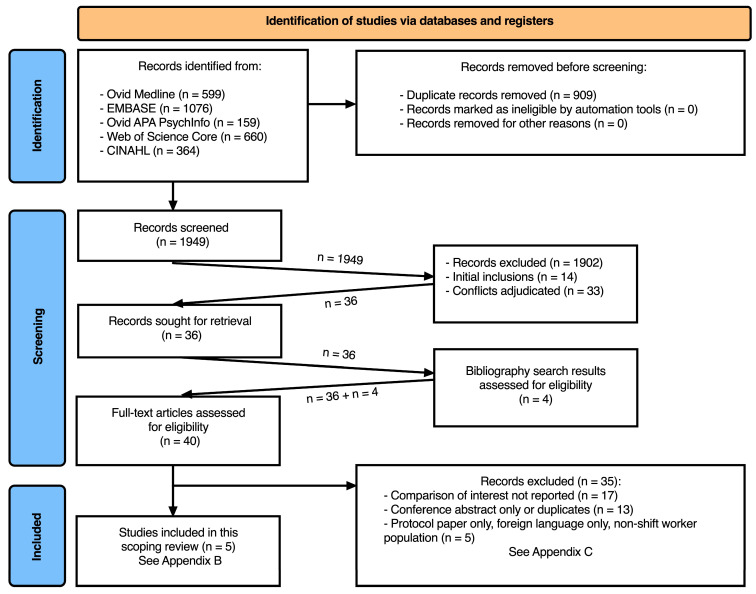
PRISMA flow diagram.

Our search strategy yielded 1949 records (titles and abstracts) after deduplication, of which 36 were retained for full-text review (Figure 1). Inter-rater agreement at the screening phase was moderate (Kappa = 0.43) and comparable to the agreement reported in previous reviews [45,46]. We searched bibliographies of all 36 retained publications and identified an additional four potentially relevant articles. In total, 40 full-text articles were evaluated against our P.I.C.O. criteria. Five articles were retained, describing three observational and two experimental research studies that targeted shift workers and examined non-invasive indicators of endothelial function in relation to the exposure to night shift work. However, none of these five studies addressed the comparison of interest: the effect of napping/sleeping during night shift work (compared to no napping/sleeping) on the non-invasive indicators of endothelial function. We charted the key findings from these five ancillary articles [28,47,48,49,50], which can be accessed in Appendix B. Below, we describe these studies along with the research gaps. In total, we excluded 35 articles out of the 40 assessed during the full-text review that did not target shift workers or failed to address multiple elements of our P.I.C.O. The reasons given for exclusion appear in Appendix C. All the publications reviewed as part of the bibliography search were excluded.

### 3.1. Description of Populations Studied

We identified five unique ancillary studies with a cumulative study sample of n = 110 individuals, of which 87% were shift workers [28,47,48,49,50]. Amir and colleagues enrolled 30 healthy physicians working at a hospital in Israel [28]. Among the enrolled, 22 were physician residents (17 internal medicine and five surgery) and eight fellows (six cardiology, one gastroenterology, and one hematology). Garu and colleagues enrolled 13 hospital workers with night duty working at a hospital in Japan [47]. This group included six men and seven women. Tarzia and colleagues recruited 20 cardiology trainees from a university in Italy [48]. This group included nine males and 11 females. Wehrens and associates enrolled 11 male shift workers and 14 non-shift workers between the ages of 25 and 45 years in the United Kingdom [49]. Zheng and colleagues enrolled 22 internal medicine residents in the U.S., of which seven were women [50]. The participant demographics reported for all the retained studies describe the participants as generally healthy and absent of medical conditions (e.g., hypertension) that may impact endothelial function following the exposure to night shift work.

### 3.2. Description of Study Design and Exposure

Three studies used a prospective observational study design [28,47,48], while two used a randomized crossover study design [49,50]. In the observational designs [28,47,48], investigators obtained the baseline measures of endothelial function prior to a standard daylight shift. The follow-up measures of endothelial function were obtained after the exposure to night shift work, most often in the morning hours post-night shift. In the one laboratory-based study, Zheng and colleagues required participants to abstain from caffeine during the study protocol and then performed two assessments of endothelial function in random order [50]. One assessment was performed after 1 p.m., following a 30 h extended shift, and the second was obtained after 1 p.m., following a 6 h daylight shift (7 a.m.–1 p.m.).

Our intervention/exposure of interest was napping/sleeping during night shift work. One study measured sleep with a questionnaire completed after night shift work [28]. Zheng and colleagues required subjects record their sleep hours during shift work on a paper-based diary and return these diaries during the post-shift assessments of endothelial function [50]. Two studies provided little or no detailed description of the methods used to document sleep during night shift work [47,48]. One laboratory-based study monitored sleep with actigraphy and polysomnography [49].

The study by Amir and colleagues reported 50% (n = 15) of participants obtaining ≥3 h of sleep during a 24 h shift and another 50% (n = 15) of participants obtaining < 3 h of sleep during the shift [28]. In the study by Garu and associates, the participants reported a mean of 2.3 h (SD1.0) of sleep during nighttime work (notably, the hours-of-work duration is not reported) [47]. Tarzia and colleagues reported 55% of participants (n = 11) obtaining <4 h of sleep during night shift work and 45% (n = 9) obtaining >4 h of sleep [48]. Zheng and colleagues reported participants obtaining an average of 0.3 h of sleep (IQR 0.0–1.5) during an extended shift of 30 h [50]. In these studies, the timing of sleep was not reported. In the laboratory-based study by Wehrens and colleagues, the participants were kept in the lab for 4 consecutive days that included a 30.5 h period of sleep deprivation [49]. The protocol allowed participants to sleep before and after the extended period of wakefulness, but not during.

### 3.3. Description of Comparisons

None of the retained studies included the comparison of interest, which was napping or sleeping during night shift work (intervention) versus no napping or no sleep during night shift work (comparison).

### 3.4. Description of Outcome Measures

Four studies used non-invasive FMD to measure endothelial function [28,48,49,50], whereas one study used the EndoPAT device (ZOLL^®^ Intamar^®^, Atlanta, GA, USA) [47]. The lower levels of FMD, expressed as a percentage, suggest impaired endothelial function. Of the four studies using FMD, one performed the FMD measurements at 1 p.m., after a traditional daylight shift, and again at 1 p.m., after a 30 h extended shift [50]. Authors reported impaired FMD following the extended shift when compared to the measures after the daylight shift (*p* < 0.05) [50]. Two studies performed two different FMD measurements in the morning (e.g., 8–9/10 a.m.), with one occurring after a night of normal sleep and again after a night shift [28,48]. Both studies showed lower FMD after a night shift compared to FMD assessed after a night of normal sleep (*p* < 0.05) [28,48]. One study performed two FMD measurements per day (once in the morning and once in evening) during a 4-day long laboratory protocol [49]. Investigators focused their analyses on the differences between shift workers and non-shift workers and detected no differences between these groups for any of the measures [49].

Two studies performed a single FMD measurement per examination session (e.g., once after a night shift) [28,50], whereas two used repeated measures [48,49]. In addition to the standard FMD measurements, two studies involved additional Nitrate-Mediated Dilation (NMD) measurements, with one study using a 25 mcg sublingual glyceryl trinitrate dose protocol [48], and one study using a 400 mcg glyceryl trinitrate sublingual spray [28]. Two studies that used FMD reported applying the ultrasound probe to the antecubital fossa and using a reactive hyperemia protocol with a brachial-placed BP cuff inflated to 200–300 mmHg for 5 min or longer [48,49]. Amir and colleagues positioned the BP cuff on the forearm [28]. Three studies reported that prior to performing the FMD measurements, the participants were placed in a supine position following a 5 to 10 min rest [28,48,49]. Three studies reported that expert or highly qualified/experienced operators performed the FMD measurements [28,48,49]. Zheng and colleagues did not report the details of their FMD protocol [50].

Garu and colleagues used the EndoPAT device for the assessment of endothelial function [47]. The EndoPAT device evaluates plethysmographic changes in micro-circulation (arterial pulse pressure/tonometry), often in the index finger before, during, and after a brief period (i.e., 5 min) of occlusion with a brachial-positioned BP cuff. The EndoPAT reported outcome is the Reactive Hyperemia Index (RHI). Previous research suggests RHI values lower than 1.67 as abnormal or are a pre-clinical indicator of endothelial dysfunction [51]. Garu and colleagues reported that study participants were assessed with the EndoPAT a total of six times (three measures before a daylight shift and three measures after a night shift) [47]. All participants were placed in a seated position with their hands at heart level during the measurements, the probes were placed on the index finger, and the BP cuff was placed on the upper arm and inflated to at least 200 mmHg during the occlusion phase [47]. The reported findings show no differences between the pre-daylight shift and post-night shift measures (*p* > 0.05) [47].

## 4. Discussion

The major findings from this scoping review include (1) previous research which suggests that the exposure to night shift work has an acute (post-night shift), negative impact on endothelial function; (2) among the studies reviewed, most involved small samples of shift workers and widely variable protocols; and (3) there is an absence of research investigating the impact of napping during night shifts on the non-invasive measures of endothelial function.

The implications of our findings for researchers and individuals concerned with or seeking to operationalize the ILO’s “Future of Work” policy suggestions and/or NIOSH’s Future of Work Initiative, or NIOSH’s Strategic Plan, is two-fold. First, researchers may use our findings to decide whether the current evidence is adequate to support the use of non-invasive assessments of endothelial function when investigating work scheduling (organizational design) and its impact on the risk of CVD. Second, researchers and policy makers must decide whether it is appropriate to invest in new/novel measurement tools and techniques for future use, like the non-invasive EndoPAT device, because these devices and approaches have the potential to assess and quantify risks at an earlier stage in disease progression compared to the current indicators like BP. How researchers and policy makers respond to these questions will have a direct impact on the attention given to the ILO’s “Future of Work” efforts and NIOSH’s Future of Work Initiative and Strategic Plan [25,35,36].

While a sizeable gap in research is identified, our scoping review uncovers opportunities for future investigations that could shed much needed light on the impact that night shift work has on the key indicators of shift worker cardiovascular health and if evidence-based interventions like napping have a clinically meaningful effect. A specific opportunity for future research is, in an adequately powered study, the comparison of napping or sleeping during night shift work versus no napping or no sleeping. This or related studies would test interventions that could be implemented in shift work occupations to improve occupational health and safety. Investigators pursuing such opportunities would directly address numerous goals outlined by leading workplace health and safety organizations like NIOSH (e.g., Intermediate Goals 1.10, 1.11, 1.13, and 7.17, and 7.1–7.12, 7.14, 7.5, 7.6, and 7.10 of the NIOSH Strategic Plan for 2019–2026) [35,36].

Numerous epidemiological studies show a consistent and compelling relationship between the years of exposure to shift work and CVD risk [2,3,4,5]. Interest among researchers in monitoring endothelial function in relation to CVD risk is substantial and will likely grow because dysfunction can be detected non-invasively and before clinical symptoms of CVD manifest and require intervention (i.e., hypertension) [14,15]. In short, monitoring endothelial function in relation to the exposure to night shift work gives researchers and clinicians targets for intervention and opportunities to assess intervention efficacy and effectiveness [13,14,29]. The findings from our scoping review show that while there is likely great interest and promise in monitoring endothelial function in relation to night shift work, the depth and breadth of existing research is far from comprehensive. Recent research demonstrates the benefits of napping during night shifts on the key indicators of CVD risk (i.e., BP) [27,30,52]. However, in this scoping review, we failed to identify a similar line of research exploring the potential benefits of night shift naps on endothelial function. While surprising, our findings suggest this is a prime area for further investigation.

Additional studies exploring the impact of night shifts on endothelial function, and the potential beneficial effects of napping (for example), could benefit from adopting rigorous experimental study designs. Most of the studies highlighted in this review were observational and the vast majority relied on one approach to measuring endothelial function (i.e., Flow Mediated Dilation). The reproducibility of FMD measures has been questioned [41]. The test results are highly dependent on technician skill and interrater agreement can be low [18,41,53,54]. Other non-invasive tools exist (i.e., the EndoPAT device) and offer opportunities to limit the need for highly trained technicians. The EndoPAT device is innovative, yet its utility has been questioned given the findings in previous research showing a low correlation with FMD [55]. Regardless of the measurement device used, investigators can improve the quality of future research by using adequately powered studies and experimental designs such as within-subject randomized crossover trials. Crossover designs are appropriate for testing the effect of interventions like napping during night shifts because the response of interest in each condition is often proximate to the exposure, where the exposure or intervention of interest does not permanently alter the study outcomes and the power is maximized with the within-subject comparisons [56].

Future studies that target and enroll shift workers will build on existing research, which make a meaningful contribution to the literature and provide stakeholders with coveted direct evidence. The studies of non-shift workers are informative, yet these data are indirect and are often downgraded in terms of the quality of evidence when collated with other studies [57]. These data are downgraded because targeted populations (i.e., shift workers) can differ from non-shift workers in meaningful ways, such as in chronotype [58]. These differences may have a clinically meaningful impact on the outcomes of interest (i.e., sleep duration) [59]. Thus, the studies focused on shift workers or shift work schedules stand a better chance of adding to the literature when enrolling shift workers as study subjects than studies with alternative populations.

### Strengths and Limitations

Common limitations of scoping reviews include incomplete database searches, the exclusion of research librarians designing search strategies, incomplete descriptions of the search and screening methods, limited details on the information abstracted from the retained literature, and variable methods for the synthesis of results [37,40]. We addressed these limitations in advance by (1) searching multiple databases and outlining the details of our search strategies in Appendix A; (2) our team included an experienced medical research librarian (RSM) with expertise in developing and refining search strategies; and (3) as prescribed [40], we documented the key findings in tables and provided a detailed, narrative, and descriptive summary of the results. We strengthened our methodology by searching the bibliographies of the retained literature to identify potentially relevant research. Finally, we adhered to recent guidance from the Preferred Reporting Items for the Systematic Reviews and Meta-Analyses extension for Scoping Reviews to ensure transparency and the opportunity for others to replicate our approach [40]. Despite these strengths, our study is limited like other reviews are limited, by (1) excluding the gray (unpublished) literature, and (2) the judgment of our investigators at multiple phases of our review. Specifically, our decisions and judgment to include or exclude any literature may differ from others; therefore, our findings may differ from other investigators.

## 5. Conclusions

While there is some evidence of an effect of night shift work on non-invasive indicators of endothelial function, this evidence is incomplete, limited to small samples of shift workers, and is mostly restricted to one measurement technique. In addition, there is no identifiable research investigating the potential benefits of napping during night shift work on the non-invasive measures of endothelial function. The limitations identified in this scoping review are also opportunities for future research that may have a meaningful impact on shift worker health and wellbeing if adequately powered and inclusive of shift workers as study subjects—providing much needed rigor in the study design and direct evidence to those who employ shift workers.

## Data Availability

Requests of the data reviewed in full-text format for this scoping literature review may be sent to the corresponding author.

## Appendix A. Search Strategy

### Appendix A.1. Duplicate Removal

**Duplicates Removed—All Databases**						
	**Ovid Medline**	**Embase.com**	**Ovid APA PsycInfo**	**Web of Science**	**EBSCOhost CINAHL**	**Totals**
Total retrieved	599	1076	159	660	364	2858
Total duplicates removed	1	351	50	360	147	909
Remaining citations	598	725	109	300	217	1949

### Appendix A.2. Search Strategies

Searches run 8 September 2022

Ovid Medline	11
Embase.com	11
Ovid APA PsycInfo	14
Web of Science Core Collection	16
EBSCOhost CINAHL	18

### Appendix A.3. Ovid Medline

**#**	**Medline Searches**	**Results**
1	Atherosclerosis/or Brachial Artery/or C-Reactive Protein/or Carotid Arteries/or Carotid Intima-Media Thickness/or Computed Tomography Angiography/or Coronary Angiography/or Coronary Circulation/or Endothelium, Vascular/or Fibrinolysis/or hyperemia/or Hyperhomocysteinemia/or Interleukin-6/or Manometry/or Nitric Oxide/or pulse wave analysis/or Tunica Intima/or vascular stiffness/or vasodilation/or (atherosclerosis or C-reactive protein or cIMT or carotid intima-media thickness or carotid ultrasound or coronary angiography or crp or EndoPat or endothelial or endothelium or flow mediated dilation or FMD or hsCRP or hs-CRP or hyperemia or il6 or il-6 or interleukin-6 or intima media thickness or nitric oxide or NO concentration or PAT or peripheral arterial tone or plethysmography or pulse wave or RHI or rh-pat or sphygmomanometer$ or tonometry or vasodilation or vascular function or vascular tone).ti,ab,kf.	1,141,024
2	occupational stress/or “Personnel Staffing and Scheduling”/or Shift Work Schedule/or work/or Work Schedule Tolerance/or (call schedule or consecutive hours or consecutive shifts or continuous work or day shift or duty hour or duty hours or evening shift or evening shifts or evening work or extended shift or extended shifts or fixed shift or fixed shifts or hour shift or hour shifts or morning shift or morning shifts or night call or night calls or night duty or night float or night schedule or night schedules or night shift or night shifts or night work or night worker$ or nightshift or nightwork or nightworker$ or nocturnal work or on-call or oncall or overnight shift or overnight shifts or (overtime adj2 work) or overtime work or rostering or rotating roster or rotating schedules or rotating shift$ or rotating work or rotation schedule or rotation schedules or rotation system or scheduling or shift cycle or shift duration$ or (shift$ adj3 h$) or shift length or shift rota or shift rotas or shift rotation or shift rotations or shift schedule or shift schedules or shift system or shift systems or shift work or shift worker$ or shiftwork or shiftworker$ or shiftworking or split-shift or staffing or work hours or work schedule or work schedules or work shift or work shifts or (work$ adj3 night$) or (work$ adj3 shift$) or working hours).ti,ab,kf.	93,366
3	1 and 2	622
4	limit 3 to yr = “1980-current”	599

### Appendix A.4. Embase.com

**#**	**Embase Searches**	**Results**
#6	#4 NOT #5	752
#5	35977673:ui OR 35696538:ui OR 35851722:ui OR 35863903:ui OR 34988862:ui OR 35758140:ui OR 35095034:ui OR 35064366:ui OR 36052588:ui OR 35879723:ui OR 35800539:ui OR 35765524:ui OR 35735819:ui OR 35316523:ui OR 35287740:ui OR 35213999:ui OR 35186577:ui OR 35932568:ui OR 35445826:ui OR 35792379:ui OR 35831532:ui OR 35822159:ui OR 35674875:ui OR 35051991:ui OR 34690250:ui OR 34232788:ui OR 35439780:ui OR 34599632:ui OR 35418421:ui OR 34487198:ui OR 35260626:ui OR 35145252:ui OR 34767077:ui OR 35226994:ui OR 35206173:ui OR 34737152:ui OR 34219158:ui OR 34849588:ui OR 34928816:ui OR 34413210:ui OR 34904496:ui OR 33622782:ui OR 33834170:ui OR 33748539:ui OR 34824802:ui OR 34104596:ui OR 33995141:ui OR 33841183:ui OR 34815273:ui OR 34125153:ui OR 33813968:ui OR 34948768:ui OR 35377986:ui OR 35192491:ui OR 34488150:ui OR 34551112:ui OR 33491873:ui OR 34251957:ui OR 34722765:ui OR 34579658:ui OR 33201246:ui OR 33558966:ui OR 33969126:ui OR 34018403:ui OR 34575881:ui OR 34353630:ui OR 34166137:ui OR 34102599:ui OR 34096425:ui OR 34050795:ui OR 33852334:ui OR 33810210:ui OR 33781135:ui OR 33596867:ui OR 33550437:ui OR 32697336:ui OR 33511906:ui OR 33487626:ui OR 33401929:ui OR 33268668:ui OR 33039210:ui OR 32895023:ui OR 32265417:ui OR 32766566:ui OR 32695969:ui OR 32617471:ui OR 32596013:ui OR 33110496:ui OR 32850531:ui OR 32842687:ui OR 32819178:ui OR 32698223:ui OR 33584665:ui OR 33317454:ui OR 33297648:ui OR 33271884:ui OR 33269898:ui OR 33083827:ui OR 33059619:ui OR 33042933:ui OR 32970584:ui OR 32926243:ui OR 32866895:ui OR 32847449:ui OR 32722586:ui OR 32697443:ui OR 32690382:ui OR 32665209:ui OR 31780790:ui OR 31874903:ui OR 32559356:ui OR 32521961:ui OR 32512234:ui OR 32498373:ui OR 32468939:ui OR 32447888:ui OR 32432519:ui OR 32274737:ui OR 32248269:ui OR 32242280:ui OR 32212409:ui OR 32174353:ui OR 32174268:ui OR 32138974:ui OR 32114501:ui OR 32097908:ui OR 32079428:ui OR 32046214:ui OR 31982412:ui OR 31963313:ui OR 31916412:ui OR 31882159:ui OR 31857218:ui OR 31845385:ui OR 31838638:ui OR 31828843:ui OR 31773540:ui OR 31764604:ui OR 31601511:ui OR 31599473:ui OR 31550699:ui OR 31352027:ui OR 31240398:ui OR 31155486:ui OR 31067130:ui OR 30765608:ui OR 30668286:ui OR 31814938:ui OR 31543966:ui OR 30800423:ui OR 30335595:ui OR 32308892:ui OR 31869395:ui OR 31819324:ui OR 31662399:ui OR 31319746:ui OR 31284736:ui OR 31232052:ui OR 31205209:ui OR 31185996:ui OR 31178469:ui OR 31116760:ui OR 31114965:ui OR 31077613:ui OR 31068499:ui OR 30979369:ui OR 30905726:ui OR 30887620:ui OR 30796476:ui OR 30759884:ui OR 30707727:ui OR 30649009:ui OR 30615593:ui OR 30598150:ui OR 30566363:ui OR 30277854:ui OR 30230913:ui OR 29796606:ui OR 31763206:ui OR 31226876:ui OR 31069130:ui OR 29896332:ui OR 30544474:ui OR 30543205:ui OR 30511769:ui OR 30389166:ui OR 30367094:ui OR 29959455:ui OR 29849876:ui OR 29794555:ui OR 29786782:ui OR 29748362:ui OR 29669967:ui OR 29615787:ui OR 29614802:ui OR 29611041:ui OR 29593172:ui OR 29467112:ui OR 29465774:ui OR 29313407:ui OR 29274045:ui OR 29130963:ui OR 29098424:ui OR 29040454:ui OR 29024973:ui OR 28963747:ui OR 28833858:ui OR 28375782:ui OR 29187945:ui OR 28814584:ui OR 28614469:ui OR 29466184:ui OR 29224662:ui OR 29224657:ui OR 29111334:ui OR 29059681:ui OR 28971637:ui OR 28924267:ui OR 28923188:ui OR 28916558:ui OR 28847906:ui OR 28692002:ui OR 28619378:ui OR 28576436:ui OR 28486343:ui OR 28442577:ui OR 28441382:ui OR 28376836:ui OR 28347188:ui OR 28257292:ui OR 28194716:ui OR 28183139:ui OR 28156158:ui OR 28069021:ui OR 27928705:ui OR 27830357:ui OR 27773752:ui OR 27687049:ui OR 27076261:ui OR 26992412:ui OR 26811352:ui OR 28674611:ui OR 28536575:ui OR 28107869:ui OR 27056124:ui OR 26467761:ui OR 28639862:ui OR 28290904:ui OR 28009392:ui OR 27984046:ui OR 27930477:ui OR 27756354:ui OR 27532642:ui OR 27511337:ui OR 27497777:ui OR 27414008:ui OR 27396538:ui OR 27379667:ui OR 27376892:ui OR 27287502:ui OR 27245641:ui OR 27207150:ui OR 27198191:ui OR 27113309:ui OR 27104805:ui OR 27081695:ui OR 27076599:ui OR 27070477:ui OR 26972869:ui OR 26940896:ui OR 26873990:ui OR 26858430:ui OR 26857924:ui OR 26614494:ui OR 26228659:ui OR 24894407:ui OR 27660715:ui OR 27095887:ui OR 26913199:ui OR 26682743:ui OR 26549394:ui OR 26487267:ui OR 26453313:ui OR 26310589:ui OR 26232234:ui OR 26215469:ui OR 26201003:ui OR 26082313:ui OR 26040512:ui OR 25915885:ui OR 25786401:ui OR 25767862:ui OR 25719984:ui OR 25362661:ui OR 25362516:ui OR 25349029:ui OR 25332463:ui OR 25323301:ui OR 25288095:ui OR 24719083:ui OR 26732516:ui OR 25821470:ui OR 24675359:ui OR 24482243:ui OR 25376409:ui OR 25187988:ui OR 25168972:ui OR 25072825:ui OR 25068265:ui OR 25046320:ui OR 25046318:ui OR 25022738:ui OR 24953089:ui OR 24944036:ui OR 24872493:ui OR 24796226:ui OR 24717852:ui OR 24569554:ui OR 24568569:ui OR 24458353:ui OR 24033699:ui OR 24025659:ui OR 23460604:ui OR 24790403:ui OR 24602344:ui OR 27024472:ui OR 24640832:ui OR 24449976:ui OR 24019174:ui OR 23964589:ui OR 23952346:ui OR 23925396:ui OR 23880723:ui OR 23758843:ui OR 23748721:ui OR 23746394:ui OR 23719543:ui OR 23696854:ui OR 23690265:ui OR 23614730:ui OR 23523470:ui OR 23499430:ui OR 23493379:ui OR 23435448:ui OR 23363434:ui OR 23363133:ui OR 23351845:ui OR 23340031:ui OR 23334903:ui OR 23324695:ui OR 23249568:ui OR 23199168:ui OR 22683167:ui OR 22082822:ui OR 23874068:ui OR 23146826:ui OR 23140921:ui OR 22918380:ui OR 22879113:ui OR 22813435:ui OR 22767870:ui OR 22716277:ui OR 22708722:ui OR 22621355:ui OR 22515415:ui OR 22494815:ui OR 22482790:ui OR 22457748:ui OR 22362851:ui OR 22347441:ui OR 22317392:ui OR 22306176:ui OR 22265452:ui OR 22261822:ui OR 22245460:ui OR 22192302:ui OR 22091987:ui OR 22062896:ui OR 21953310:ui OR 21903367:ui OR 21900367:ui OR 21455800:ui OR 22457684:ui OR 21808500:ui OR 22188900:ui OR 22117767:ui OR 22037099:ui OR 21892899:ui OR 21880287:ui OR 21797781:ui OR 21792179:ui OR 21764158:ui OR 21697626:ui OR 21672368:ui OR 21640226:ui OR 21536769:ui OR 21324067:ui OR 21297153:ui OR 21247546:ui OR 21204622:ui OR 21145027:ui OR 21128931:ui OR 21107332:ui OR 20602750:ui OR 21203463:ui OR 21141454:ui OR 21092618:ui OR 20846058:ui OR 20832020:ui OR 20740351:ui OR 20618695:ui OR 20603084:ui OR 20584358:ui OR 20507901:ui OR 20351338:ui OR 20237824:ui OR 20144696:ui OR 20106477:ui OR 20087536:ui OR 19895780:ui OR 19804986:ui OR 19736177:ui OR 19701751:ui OR 19697323:ui OR 19881951:ui OR 19902504:ui OR 19877763:ui OR 19858982:ui OR 19796569:ui OR 19670313:ui OR 19580972:ui OR 19580496:ui OR 19572477:ui OR 19410368:ui OR 19407805:ui OR 19319982:ui OR 19284605:ui OR 19242279:ui OR 19215924:ui OR 19199360:ui OR 19184266:ui OR 19165621:ui OR 18307781:ui OR 17393165:ui OR 18819995:ui OR 19463384:ui OR 19400524:ui OR 19181016:ui OR 19145816:ui OR 19096491:ui OR 19014781:ui OR 19010843:ui OR 18815150:ui OR 18628697:ui OR 18391652:ui OR 18321520:ui OR 18310774:ui OR 18297195:ui OR 18266977:ui OR 18240990:ui OR 18202011:ui OR 17561943:ui OR 17554539:ui OR 19238648:ui OR 18273976:ui OR 18217257:ui OR 17900004:ui OR 17606728:ui OR 17545548:ui OR 17497974:ui OR 17485874:ui OR 17459681:ui OR 17458650:ui OR 17364583:ui OR 17329220:ui OR 17285221:ui OR 17234361:ui OR 17097166:ui OR 16710697:ui OR 16377413:ui OR 16954481:ui OR 16877730:ui OR 16827808:ui OR 16685440:ui OR 16598598:ui OR 16567564:ui OR 16428928:ui OR 16435433:ui OR 16388726:ui OR 16301095:ui OR 16241923:ui OR 16204432:ui OR 16046611:ui OR 15930363:ui OR 15814654:ui OR 15805338:ui OR 15701874:ui OR 15565458:ui OR 15623642:ui OR 15673094:ui OR 15640778:ui OR 15615476:ui OR 15457367:ui OR 15347779:ui OR 15276596:ui OR 15218747:ui OR 15133229:ui OR 15050508:ui OR 15036659:ui OR 15005678:ui OR 14962820:ui OR 15456711:ui OR 14627895:ui OR 12960735:ui OR 12937187:ui OR 12869390:ui OR 12830447:ui OR 12821274:ui OR 12767957:ui OR 12739982:ui OR 12673734:ui OR 12552252:ui OR 12476362:ui OR 12423578:ui OR 12353236:ui OR 12222070:ui OR 12167189:ui OR 12114444:ui OR 12071548:ui OR 11850553:ui OR 11836462:ui OR 21318803:ui OR 14564902:ui OR 11902515:ui OR 11881367:ui OR 11742695:ui OR 11681035:ui OR 11487011:ui OR 11403349:ui OR 11348065:ui OR 10969807:ui OR 10914731:ui OR 10862494:ui OR 10766175:ui OR 10698245:ui OR 10593632:ui OR 10593631:ui OR 10545553:ui OR 10212162:ui OR 12793961:ui OR 9730580:ui OR 9669248:ui OR 9233740:ui OR 9185648:ui OR 9166272:ui OR 9081265:ui OR 8800164:ui OR 8773915:ui OR 8697140:ui OR 8777753:ui OR 8525226:ui OR 8520862:ui OR 7757384:ui OR 7536682:ui OR 7803432:ui OR 7740865:ui OR 8402734:ui OR 8401260:ui OR 8260274:ui OR 8214761:ui OR 8128449:ui OR 8093344:ui OR 7682469:ui OR 1596568:ui OR 1490974:ui OR 1479768:ui OR 1290685:ui OR 1992786:ui OR 1991671:ui OR 1853307:ui OR 1825675:ui OR 1818784:ui OR 10107102:ui OR 2722227:ui OR 3348842:ui OR 2977333:ui OR 2443046:ui OR 3995694:ui OR 3988530:ui OR 3842823:ui OR 6732347:ui OR 6437717:ui OR 6242472:ui OR 6679840:ui OR 6345701:ui OR 6085930:ui OR 6982601:ui OR 7465844:ui	551
#4	#1 AND #2 AND [1980–2022]/py	1076
#3	#1 AND #2	1094
#2	‘afternoon shift’/de OR ‘evening shift’/de OR ‘morning shift’/de OR ‘night shift’/de OR ‘night shift worker’/de OR ‘rotating shift’/de OR ‘rotating shift worker’/de OR ‘shift schedule’/de OR ‘shift work’/de OR ‘shift worker’/de OR ‘work’/de OR ‘work schedule’/de OR ‘call schedule’:ti,ab,kw OR ‘consecutive hours’:ti,ab,kw OR ‘consecutive shifts’:ti,ab,kw OR ‘continuous work’:ti,ab,kw OR ‘day shift’:ti,ab,kw OR ‘duty hour’:ti,ab,kw OR ‘duty hours’:ti,ab,kw OR ‘evening shift’:ti,ab,kw OR ‘evening shifts’:ti,ab,kw OR ‘evening work’:ti,ab,kw OR ‘extended shift’:ti,ab,kw OR ‘extended shifts’:ti,ab,kw OR ‘fixed shift’:ti,ab,kw OR ‘fixed shifts’:ti,ab,kw OR ‘hour shift’:ti,ab,kw OR ‘hour shifts’:ti,ab,kw OR ‘morning shift’:ti,ab,kw OR ‘morning shifts’:ti,ab,kw OR ‘night call’:ti,ab,kw OR ‘night calls’:ti,ab,kw OR ‘night duty’:ti,ab,kw OR ‘night float’:ti,ab,kw OR ‘night schedule’:ti,ab,kw OR ‘night schedules’:ti,ab,kw OR ‘night shift’:ti,ab,kw OR ‘night shifts’:ti,ab,kw OR ‘night work’:ti,ab,kw OR ‘night worker’:ti,ab,kw OR ‘night workers’:ti,ab,kw OR nightshift:ti,ab,kw OR nightwork:ti,ab,kw OR nightworker*:ti,ab,kw OR ‘nocturnal work’:ti,ab,kw OR ‘on-call’:ti,ab,kw OR ‘oncall’:ti,ab,kw OR ‘overnight shift’:ti,ab,kw OR ‘overnight shifts’:ti,ab,kw OR ((overtime NEAR/2 work):ti,ab,kw) OR ‘overtime work’:ti,ab,kw OR rostering:ti,ab,kw OR ‘rotating roster’:ti,ab,kw OR ‘rotating schedules’:ti,ab,kw OR ‘rotating shift’:ti,ab,kw OR ‘rotating shifts’:ti,ab,kw OR ‘rotating work’:ti,ab,kw OR ‘rotation schedule’:ti,ab,kw OR ‘rotation schedules’:ti,ab,kw OR ‘rotation system’:ti,ab,kw OR ‘shift cycle’:ti,ab,kw OR ‘shift duration’:ti,ab,kw OR ‘shift durations’:ti,ab,kw OR ((shift* NEAR/3 h*):ti,ab,kw) OR ‘shift length’:ti,ab,kw OR ‘shift rota’:ti,ab,kw OR ‘shift rotas’:ti,ab,kw OR ‘shift rotation’:ti,ab,kw OR ‘shift rotations’:ti,ab,kw OR ‘shift schedule’:ti,ab,kw OR ‘shift schedules’:ti,ab,kw OR ‘shift system’:ti,ab,kw OR ‘shift systems’:ti,ab,kw OR ‘shift work’:ti,ab,kw OR ‘shift worker’:ti,ab,kw OR ‘shift workers’:ti,ab,kw OR shiftwork:ti,ab,kw OR shiftworker:ti,ab,kw OR shiftworkers:ti,ab,kw OR shiftworking:ti,ab,kw OR ‘split-shift’:ti,ab,kw OR ‘work hours’:ti,ab,kw OR ‘work schedule’:ti,ab,kw OR ‘work schedules’:ti,ab,kw OR ‘work shift’:ti,ab,kw OR ‘work shifts’:ti,ab,kw OR ((work* NEAR/3 night*):ti,ab,kw) OR ((work* NEAR/3 shift*):ti,ab,kw) OR ‘working hours’:ti,ab,kw	86,045
#1	‘arterial wall thickness’/de OR ‘artery dilatation’/de OR ‘artery intima proliferation’/de OR ‘atherosclerosis’/de OR ‘brachial artery’/de OR ‘c reactive protein’/de OR ‘carotid artery’/de OR ‘carotid artery bifurcation’/de OR ‘carotid atherosclerosis’/de OR ‘carotid intima-media thickness’/de OR ‘common carotid artery’/de OR ‘computed tomographic angiography’/de OR ‘coronary angiography’/de OR ‘coronary artery atherosclerosis’/de OR ‘coronary artery blood flow’/de OR ‘endothelial dysfunction’/de OR ‘fibrinolysis’/de OR ‘fractional exhaled nitric oxide’/de OR ‘hyperemia’/de OR ‘hyperhomocysteinemia’/de OR ‘interleukin 6’/de OR ‘intima’/de OR ‘manometry’/de OR ‘nitric oxide’/de OR ‘pulse wave’/de OR ‘pulse wave velocity’/de OR ‘arterial stiffness’/de OR ‘vascular endothelium’/de OR ‘vasodilatation’/de OR atherosclerosis:ti,ab,kw OR ‘c-reactive protein’:ti,ab,kw OR cimt:ti,ab,kw OR ‘carotid intima-media thickness’:ti,ab,kw OR ‘carotid ultrasound’:ti,ab,kw OR ‘computed tomography angiography’:ti,ab,kw OR ‘coronary angiography’:ti,ab,kw OR crp:ti,ab,kw OR endopat:ti,ab,kw OR endothelial:ti,ab,kw OR endothelium:ti,ab,kw OR ‘flow mediated dilation’:ti,ab,kw OR fmd:ti,ab,kw OR hscrp:ti,ab,kw OR ‘hs-crp’:ti,ab,kw OR hyperemia:ti,ab,kw OR il6:ti,ab,kw OR ‘il-6’:ti,ab,kw OR ‘interleukin-6’:ti,ab,kw OR ‘intima media thickness’:ti,ab,kw OR ‘nitric oxide’:ti,ab,kw OR ‘no concentration’:ti,ab,kw OR pat:ti,ab,kw OR ‘peripheral arterial tone’:ti,ab,kw OR plethysmography:ti,ab,kw OR ‘pulse wave’:ti,ab,kw OR rhi:ti,ab,kw OR ‘rh-pat’:ti,ab,kw OR sphygmomanometer*:ti,ab,kw OR tonometry:ti,ab,kw OR ‘vascular function’:ti,ab,kw OR ‘vascular tone’:ti,ab,kw OR vasodilation:ti,ab,kw	1,868,244

### Appendix A.5. Ovid APA PsycInfo

**#**	**PsycInfo Searches**	**Results**
1	“arteries (anatomy)”/or Atherosclerosis/or blood coagulation/or Carotid Arteries/or Interleukins/or Nitric Oxide/or Proteins/or Vasodilation/or (atherosclerosis or C-reactive protein or cIMT or carotid intima-media thickness or carotid ultrasound or coronary angiography or crp or EndoPat or endothelial or endothelium or flow mediated dilation or FMD or hsCRP or hs-CRP or hyperemia or il6 or il-6 or interleukin-6 or intima media thickness or nitric oxide or NO concentration or PAT or peripheral arterial tone or plethysmography or pulse wave or RHI or rh-pat or sphygmomanometer$ or tonometry or vascular function or vascular tone or vasodilation).tw.	59,975
2	Occupational Stress/or Work Rest Cycles/or Work Scheduling/or work week length/or Workday Shifts/or Working Conditions/or (call schedule or consecutive hours or consecutive shifts or continuous work or day shift or duty hour or duty hours or evening shift or evening shifts or evening work or extended shift or extended shifts or fixed shift or fixed shifts or hour shift or hour shifts or morning shift or morning shifts or night call or night calls or night duty or night float or night schedule or night schedules or night shift or night shifts or night work or night worker$ or nightshift or nightwork or nightworker$ or nocturnal work or on-call or oncall or overnight shift or overnight shifts or (overtime adj2 work) or overtime work or rostering or rotating roster or rotating schedules or rotating shift$ or rotating work or rotation schedule or rotation schedules or rotation system or scheduling or shift cycle or shift duration$ or (shift$ adj3 h$) or shift length or shift rota or shift rotas or shift rotation or shift rotations or shift schedule or shift schedules or shift system or shift systems or shift work or shift worker$ or shiftwork or shiftworker$ or shiftworking or split-shift or staffing or work hours or work schedule or work schedules or work shift or work shifts or (work$ adj3 night$) or (work$ adj3 shift$) or working hours).tw.	65,560
3	1 and 2	159
4	limit 3 to yr = “1980-current”	159
5	(“35977673” or “35696538” or “35851722” or “35863903” or “34988862” or “35758140” or “35095034” or “35064366” or “36052588” or “35879723” or “35800539” or “35765524” or “35735819” or “35316523” or “35287740” or “35213999” or “35186577” or “35932568” or “35445826” or “35792379” or “35831532” or “35822159” or “35674875” or “35051991” or “34690250” or “34232788” or “35439780” or “34599632” or “35418421” or “35418421” or “34487198” or “35260626” or “35145252” or “34767077” or “35226994” or “35206173” or “34737152” or “34219158” or “34849588” or “34928816” or “34413210” or “34904496” or “33622782” or “33834170” or “33748539” or “34824802” or “34104596” or “33995141” or “33841183” or “34815273” or “34125153” or “33813968” or “34948768” or “35377986” or “35192491” or “34488150” or “34551112” or “33491873” or “34251957” or “34722765” or “34579658” or “33201246” or “33558966” or “33969126” or “34018403” or “34575881” or “34353630” or “34166137” or “34102599” or “34096425” or “34050795” or “33852334” or “33810210” or “33781135” or “33596867” or “33550437” or “32697336” or “33511906” or “33487626” or “33401929” or “33268668” or “33039210” or “32895023” or “32265417” or “32766566” or “32695969” or “32617471” or “32596013” or “33110496” or “32850531” or “32842687” or “32819178” or “32698223” or “33584665” or “33317454” or “33297648” or “33271884” or “33269898” or “33083827” or “33059619” or “33042933” or “32970584” or “32926243” or “32866895” or “32847449” or “32722586” or “32697443” or “32690382” or “32665209” or “31780790” or “31874903” or “32559356” or “32521961” or “32512234” or “32498373” or “32468939” or “32447888” or “32432519” or “32274737” or “32248269” or “32242280” or “32212409” or “32174353” or “32174268” or “32138974” or “32114501” or “32097908” or “32079428” or “32046214” or “31982412” or “31963313” or “31916412” or “31882159” or “31857218” or “31845385” or “31838638” or “31828843” or “31773540” or “31764604” or “31601511” or “31599473” or “31550699” or “31352027” or “31240398” or “31155486” or “31067130” or “30765608” or “30668286” or “31814938” or “31543966” or “30800423” or “30335595” or “32308892” or “31869395” or “31819324” or “31662399” or “31319746” or “31284736” or “31232052” or “31205209” or “31185996” or “31178469” or “31116760” or “31114965” or “31077613” or “31068499” or “30979369” or “30905726” or “30887620” or “30796476” or “30759884” or “30707727” or “30649009” or “30615593” or “30598150” or “30566363” or “30277854” or “30230913” or “29796606” or “31763206” or “31226876” or “31069130” or “29896332” or “30544474” or “30543205” or “30511769” or “30389166” or “30367094” or “29959455” or “29849876” or “29794555” or “29786782” or “29748362” or “29669967” or “29615787” or “29614802” or “29611041” or “29593172” or “29467112” or “29465774” or “29313407” or “29274045” or “29130963” or “29098424” or “29040454” or “29024973” or “28963747” or “28833858” or “28375782” or “29187945” or “28814584” or “28614469” or “29466184” or “29224662” or “29224657” or “29111334” or “29059681” or “28971637” or “28924267” or “28923188” or “28916558” or “28847906” or “28692002” or “28619378” or “28576436” or “28486343” or “28442577” or “28441382” or “28376836” or “28347188” or “28257292” or “28194716” or “28183139” or “28156158” or “28069021” or “27928705” or “27830357” or “27773752” or “27687049” or “27076261” or “26992412” or “26811352” or “28674611” or “28536575” or “28107869” or “27056124” or “26467761” or “28639862” or “28290904” or “28009392” or “27984046” or “27930477” or “27756354” or “27532642” or “27511337” or “27497777” or “27414008” or “27396538” or “27379667” or “27376892” or “27287502” or “27245641” or “27207150” or “27198191” or “27113309” or “27104805” or “27081695” or “27076599” or “27070477” or “26972869” or “26940896” or “26873990” or “26858430” or “26857924” or “26614494” or “26228659” or “24894407” or “27660715” or “27095887” or “26913199” or “26682743” or “26549394” or “26487267” or “26453313” or “26310589” or “26232234” or “26215469” or “26201003” or “26082313” or “26040512” or “25915885” or “25786401” or “25767862” or “25719984” or “25362661” or “25362516” or “25349029” or “25332463” or “25323301” or “25288095” or “24719083” or “26732516” or “25821470” or “24675359” or “24482243” or “25376409” or “25187988” or “25168972” or “25072825” or “25068265” or “25046320” or “25046318” or “25022738” or “24953089” or “24944036” or “24872493” or “24796226” or “24717852” or “24569554” or “24568569” or “24458353” or “24033699” or “24025659” or “23460604” or “24790403” or “24602344” or “27024472” or “24640832” or “24449976” or “24019174” or “23964589” or “23952346” or “23925396” or “23880723” or “23758843” or “23748721” or “23746394” or “23719543” or “23696854” or “23690265” or “23614730” or “23523470” or “23499430” or “23493379” or “23435448” or “23363434” or “23363133” or “23351845” or “23340031” or “23334903” or “23324695” or “23249568” or “23199168” or “22683167” or “22082822” or “23874068” or “23146826” or “23140921” or “22918380” or “22879113” or “22813435” or “22767870” or “22716277” or “22708722” or “22621355” or “22515415” or “22494815” or “22482790” or “22457748” or “22362851” or “22347441” or “22317392” or “22306176” or “22265452” or “22261822” or “22245460” or “22192302” or “22091987” or “22062896” or “21953310” or “21903367” or “21900367” or “21455800” or “22457684” or “21808500” or “22188900” or “22117767” or “22037099” or “21892899” or “21880287” or “21797781” or “21792179” or “21764158” or “21697626” or “21672368” or “21640226” or “21536769” or “21324067” or “21297153” or “21247546” or “21204622” or “21145027” or “21128931” or “21107332” or “20602750” or “21203463” or “21141454” or “21092618” or “20846058” or “20832020” or “20740351” or “20618695” or “20603084” or “20584358” or “20507901” or “20351338” or “20237824” or “20144696” or “20106477” or “20087536” or “19895780” or “19804986” or “19736177” or “19701751” or “19697323” or “19881951” or “19902504” or “19877763” or “19858982” or “19796569” or “19670313” or “19580972” or “19580496” or “19572477” or “19410368” or “19407805” or “19319982” or “19284605” or “19242279” or “19215924” or “19199360” or “19184266” or “19165621” or “18307781” or “17393165” or “18819995” or “19463384” or “19400524” or “19181016” or “19145816” or “19096491” or “19014781” or “19010843” or “18815150” or “18628697” or “18391652” or “18321520” or “18310774” or “18297195” or “18266977” or “18240990” or “18202011” or “17561943” or “17554539” or “19238648” or “18273976” or “18217257” or “17900004” or “17606728” or “17545548” or “17497974” or “17485874” or “17459681” or “17458650” or “17364583” or “17329220” or “17285221” or “17234361” or “17097166” or “16710697” or “16377413” or “16954481” or “16877730” or “16827808” or “16685440” or “16598598” or “16567564” or “16428928” or “16435433” or “16388726” or “16301095” or “16241923” or “16204432” or “16046611” or “15930363” or “15814654” or “15805338” or “15701874” or “15565458” or “15623642” or “15673094” or “15640778” or “15615476” or “15457367” or “15347779” or “15276596” or “15218747” or “15133229” or “15050508” or “15036659” or “15005678” or “14962820” or “15456711” or “14627895” or “12960735” or “12937187” or “12869390” or “12830447” or “12821274” or “12767957” or “12739982” or “12673734” or “12552252” or “12476362” or “12423578” or “12353236” or “12222070” or “12167189” or “12114444” or “12071548” or “11850553” or “11836462” or “21318803” or “14564902” or “11902515” or “11881367” or “11742695” or “11681035” or “11487011” or “11403349” or “11348065” or “10969807” or “10914731” or “10862494” or “10766175” or “10698245” or “10593632” or “10593631” or “10545553” or “10212162” or “12793961” or “9730580” or “9669248” or “9233740” or “9185648” or “9166272” or “9081265” or “8800164” or “8773915” or “8697140” or “8777753” or “8525226” or “8520862” or “7757384” or “7536682” or “7803432” or “7740865” or “8402734” or “8401260” or “8260274” or “8214761” or “8128449” or “8093344” or “7682469” or “1596568” or “1490974” or “1479768” or “1290685” or “1992786” or “1991671” or “1853307” or “1825675” or “1818784” or “10107102” or “2722227” or “3348842” or “2977333” or “2443046” or “3995694” or “3988530” or “3842823” or “6732347” or “6437717” or “6242472” or “6679840” or “6345701” or “6085930” or “6982601” or “7465844”).pm.	46
6	4 not 5	120

### Appendix A.6. Web of Science Core Collection

Editions = A&HCI, ESCI, SCI-EXPANDED, SSCI

**#**	**Web of Science Searches**	**Results**
#6	#4 NOT #5	354
#5	PMID = (35977673 OR 35696538 OR 35851722 OR 35863903 OR 34988862 OR 35758140 OR 35095034 OR 35064366 OR 36052588 OR 35879723 OR 35800539 OR 35765524 OR 35735819 OR 35316523 OR 35287740 OR 35213999 OR 35186577 OR 35932568 OR 35445826 OR 35792379 OR 35831532 OR 35822159 OR 35674875 OR 35051991 OR 34690250 OR 34232788 OR 35439780 OR 34599632 OR 35418421 OR 35418421 OR 34487198 OR 35260626 OR 35145252 OR 34767077 OR 35226994 OR 35206173 OR 34737152 OR 34219158 OR 34849588 OR 34928816 OR 34413210 OR 34904496 OR 33622782 OR 33834170 OR 33748539 OR 34824802 OR 34104596 OR 33995141 OR 33841183 OR 34815273 OR 34125153 OR 33813968 OR 34948768 OR 35377986 OR 35192491 OR 34488150 OR 34551112 OR 33491873 OR 34251957 OR 34722765 OR 34579658 OR 33201246 OR 33558966 OR 33969126 OR 34018403 OR 34575881 OR 34353630 OR 34166137 OR 34102599 OR 34096425 OR 34050795 OR 33852334 OR 33810210 OR 33781135 OR 33596867 OR 33550437 OR 32697336 OR 33511906 OR 33487626 OR 33401929 OR 33268668 OR 33039210 OR 32895023 OR 32265417 OR 32766566 OR 32695969 OR 32617471 OR 32596013 OR 33110496 OR 32850531 OR 32842687 OR 32819178 OR 32698223 OR 33584665 OR 33317454 OR 33297648 OR 33271884 OR 33269898 OR 33083827 OR 33059619 OR 33042933 OR 32970584 OR 32926243 OR 32866895 OR 32847449 OR 32722586 OR 32697443 OR 32690382 OR 32665209 OR 31780790 OR 31874903 OR 32559356 OR 32521961 OR 32512234 OR 32498373 OR 32468939 OR 32447888 OR 32432519 OR 32274737 OR 32248269 OR 32242280 OR 32212409 OR 32174353 OR 32174268 OR 32138974 OR 32114501 OR 32097908 OR 32079428 OR 32046214 OR 31982412 OR 31963313 OR 31916412 OR 31882159 OR 31857218 OR 31845385 OR 31838638 OR 31828843 OR 31773540 OR 31764604 OR 31601511 OR 31599473 OR 31550699 OR 31352027 OR 31240398 OR 31155486 OR 31067130 OR 30765608 OR 30668286 OR 31814938 OR 31543966 OR 30800423 OR 30335595 OR 32308892 OR 31869395 OR 31819324 OR 31662399 OR 31319746 OR 31284736 OR 31232052 OR 31205209 OR 31185996 OR 31178469 OR 31116760 OR 31114965 OR 31077613 OR 31068499 OR 30979369 OR 30905726 OR 30887620 OR 30796476 OR 30759884 OR 30707727 OR 30649009 OR 30615593 OR 30598150 OR 30566363 OR 30277854 OR 30230913 OR 29796606 OR 31763206 OR 31226876 OR 31069130 OR 29896332 OR 30544474 OR 30543205 OR 30511769 OR 30389166 OR 30367094 OR 29959455 OR 29849876 OR 29794555 OR 29786782 OR 29748362 OR 29669967 OR 29615787 OR 29614802 OR 29611041 OR 29593172 OR 29467112 OR 29465774 OR 29313407 OR 29274045 OR 29130963 OR 29098424 OR 29040454 OR 29024973 OR 28963747 OR 28833858 OR 28375782 OR 29187945 OR 28814584 OR 28614469 OR 29466184 OR 29224662 OR 29224657 OR 29111334 OR 29059681 OR 28971637 OR 28924267 OR 28923188 OR 28916558 OR 28847906 OR 28692002 OR 28619378 OR 28576436 OR 28486343 OR 28442577 OR 28441382 OR 28376836 OR 28347188 OR 28257292 OR 28194716 OR 28183139 OR 28156158 OR 28069021 OR 27928705 OR 27830357 OR 27773752 OR 27687049 OR 27076261 OR 26992412 OR 26811352 OR 28674611 OR 28536575 OR 28107869 OR 27056124 OR 26467761 OR 28639862 OR 28290904 OR 28009392 OR 27984046 OR 27930477 OR 27756354 OR 27532642 OR 27511337 OR 27497777 OR 27414008 OR 27396538 OR 27379667 OR 27376892 OR 27287502 OR 27245641 OR 27207150 OR 27198191 OR 27113309 OR 27104805 OR 27081695 OR 27076599 OR 27070477 OR 26972869 OR 26940896 OR 26873990 OR 26858430 OR 26857924 OR 26614494 OR 26228659 OR 24894407 OR 27660715 OR 27095887 OR 26913199 OR 26682743 OR 26549394 OR 26487267 OR 26453313 OR 26310589 OR 26232234 OR 26215469 OR 26201003 OR 26082313 OR 26040512 OR 25915885 OR 25786401 OR 25767862 OR 25719984 OR 25362661 OR 25362516 OR 25349029 OR 25332463 OR 25323301 OR 25288095 OR 24719083 OR 26732516 OR 25821470 OR 24675359 OR 24482243 OR 25376409 OR 25187988 OR 25168972 OR 25072825 OR 25068265 OR 25046320 OR 25046318 OR 25022738 OR 24953089 OR 24944036 OR 24872493 OR 24796226 OR 24717852 OR 24569554 OR 24568569 OR 24458353 OR 24033699 OR 24025659 OR 23460604 OR 24790403 OR 24602344 OR 27024472 OR 24640832 OR 24449976 OR 24019174 OR 23964589 OR 23952346 OR 23925396 OR 23880723 OR 23758843 OR 23748721 OR 23746394 OR 23719543 OR 23696854 OR 23690265 OR 23614730 OR 23523470 OR 23499430 OR 23493379 OR 23435448 OR 23363434 OR 23363133 OR 23351845 OR 23340031 OR 23334903 OR 23324695 OR 23249568 OR 23199168 OR 22683167 OR 22082822 OR 23874068 OR 23146826 OR 23140921 OR 22918380 OR 22879113 OR 22813435 OR 22767870 OR 22716277 OR 22708722 OR 22621355 OR 22515415 OR 22494815 OR 22482790 OR 22457748 OR 22362851 OR 22347441 OR 22317392 OR 22306176 OR 22265452 OR 22261822 OR 22245460 OR 22192302 OR 22091987 OR 22062896 OR 21953310 OR 21903367 OR 21900367 OR 21455800 OR 22457684 OR 21808500 OR 22188900 OR 22117767 OR 22037099 OR 21892899 OR 21880287 OR 21797781 OR 21792179 OR 21764158 OR 21697626 OR 21672368 OR 21640226 OR 21536769 OR 21324067 OR 21297153 OR 21247546 OR 21204622 OR 21145027 OR 21128931 OR 21107332 OR 20602750 OR 21203463 OR 21141454 OR 21092618 OR 20846058 OR 20832020 OR 20740351 OR 20618695 OR 20603084 OR 20584358 OR 20507901 OR 20351338 OR 20237824 OR 20144696 OR 20106477 OR 20087536 OR 19895780 OR 19804986 OR 19736177 OR 19701751 OR 19697323 OR 19881951 OR 19902504 OR 19877763 OR 19858982 OR 19796569 OR 19670313 OR 19580972 OR 19580496 OR 19572477 OR 19410368 OR 19407805 OR 19319982 OR 19284605 OR 19242279 OR 19215924 OR 19199360 OR 19184266 OR 19165621 OR 18307781 OR 17393165 OR 18819995 OR 19463384 OR 19400524 OR 19181016 OR 19145816 OR 19096491 OR 19014781 OR 19010843 OR 18815150 OR 18628697 OR 18391652 OR 18321520 OR 18310774 OR 18297195 OR 18266977 OR 18240990 OR 18202011 OR 17561943 OR 17554539 OR 19238648 OR 18273976 OR 18217257 OR 17900004 OR 17606728 OR 17545548 OR 17497974 OR 17485874 OR 17459681 OR 17458650 OR 17364583 OR 17329220 OR 17285221 OR 17234361 OR 17097166 OR 16710697 OR 16377413 OR 16954481 OR 16877730 OR 16827808 OR 16685440 OR 16598598 OR 16567564 OR 16428928 OR 16435433 OR 16388726 OR 16301095 OR 16241923 OR 16204432 OR 16046611 OR 15930363 OR 15814654 OR 15805338 OR 15701874 OR 15565458 OR 15623642 OR 15673094 OR 15640778 OR 15615476 OR 15457367 OR 15347779 OR 15276596 OR 15218747 OR 15133229 OR 15050508 OR 15036659 OR 15005678 OR 14962820 OR 15456711 OR 14627895 OR 12960735 OR 12937187 OR 12869390 OR 12830447 OR 12821274 OR 12767957 OR 12739982 OR 12673734 OR 12552252 OR 12476362 OR 12423578 OR 12353236 OR 12222070 OR 12167189 OR 12114444 OR 12071548 OR 11850553 OR 11836462 OR 21318803 OR 14564902 OR 11902515 OR 11881367 OR 11742695 OR 11681035 OR 11487011 OR 11403349 OR 11348065 OR 10969807 OR 10914731 OR 10862494 OR 10766175 OR 10698245 OR 10593632 OR 10593631 OR 10545553 OR 10212162 OR 12793961 OR 9730580 OR 9669248 OR 9233740 OR 9185648 OR 9166272 OR 9081265 OR 8800164 OR 8773915 OR 8697140 OR 8777753 OR 8525226 OR 8520862 OR 7757384 OR 7536682 OR 7803432 OR 7740865 OR 8402734 OR 8401260 OR 8260274 OR 8214761 OR 8128449 OR 8093344 OR 7682469 OR 1596568 OR 1490974 OR 1479768 OR 1290685 OR 1992786 OR 1991671 OR 1853307 OR 1825675 OR 1818784 OR 10107102 OR 2722227 OR 3348842 OR 2977333 OR 2443046 OR 3995694 OR 3988530 OR 3842823 OR 6732347 OR 6437717 OR 6242472 OR 6679840 OR 6345701 OR 6085930 OR 6982601 OR 7465844)	523
#4	#1 AND #2 Timespan: 1 January1980 to 31 December 2022 (Publication Date)	660
#3	#1 AND #2	660
#2	TS = (“call schedule” OR “consecutive hours” OR “consecutive shifts” OR “continuous work” OR “day shift” OR “duty hour” OR “duty hours” OR “evening shift” OR “evening shifts” OR “evening work” OR “extended shift” OR “extended shifts” OR “fixed shift” OR “fixed shifts” OR “hour shift” OR “hour shifts” OR “job stress” OR “morning shift” OR “morning shifts” OR “night call” OR “night calls” OR “night duty” OR “night float” OR “night schedule” OR “night schedules” OR “night shift” OR “night shifts” OR “night work” OR “night worker” OR “night workers” OR nightshift OR “nightwork” OR nightworker* OR “nocturnal work” OR “occupational stress” OR “on call” OR “oncall” OR “overnight shift” OR “overnight shifts” OR (overtime NEAR/2 work) OR “overtime work” OR rostering OR “rotating roster” OR “rotating schedules” OR “rotating shift” OR “rotating shifts” OR “rotating work” OR “rotation schedule” OR “rotation schedules” OR “rotation system” OR “shift cycle” OR “shift duration” OR “shift durations” OR (shift* NEAR/3 h*) OR “shift length” OR “shift rota” OR “shift rotas” OR “shift rotation” OR “shift rotations” OR “shift schedule” OR “shift schedules” OR “shift system” OR “shift systems” OR “shift work” OR “shift worker” OR “shift workers” OR “shiftwork” OR shiftworker* OR shiftworking OR “split shift” OR “staffing” OR “work hours” OR “work schedule” OR “work schedules” OR “work shift” OR “work shifts” OR (work* NEAR/3 night*) OR (work* NEAR/3 shift*) OR “working hours”)	66,989
#1	TS = (atherosclerosis OR “brachial artery” OR “c-reactive protein” OR cIMT OR crp OR “carotid artery” OR “carotid arteries” OR “carotid intima-media thickness” OR “carotid ultrasound” OR “coronary angiography” OR “coronary circulation” OR EndoPat OR endothelial OR “endothelium” OR fibrinolysis OR “flow mediated dilation” OR FMD OR hsCRP OR “hs-CRP” OR “hyperaemia” OR “hyperemia” OR hyperhomocysteinemia OR il6 OR “il-6” OR “interleukin-6” OR “intima media thickness” OR manometry OR “nitric oxide” OR “NO concentration” OR “PAT” OR “peripheral arterial tone” OR plethysmography OR “pulse wave” OR RHI OR “rh-pat” OR sphygmomanometer* OR tonometry OR “tunica intima” OR “vascular function” OR “vascular stiffness” OR “vascular tone” OR vasodilation)	1,359,362

### Appendix A.7. EBSCOhost CINAHL

Note: no date limit was added to this search as no articles prior to 1980 were returned.

**#**	**CINAHL Searches**	**Results**
S5	S3 NOT S4	273
S4	PM (35977673 OR NLM35696538 OR NLM35851722 OR NLM35863903 OR NLM34988862 OR NLM35758140 OR NLM35095034 OR NLM35064366 OR NLM36052588 OR NLM35879723 OR NLM35800539 OR NLM35765524 OR NLM35735819 OR NLM35316523 OR NLM35287740 OR NLM35213999 OR NLM35186577 OR NLM35932568 OR NLM35445826 OR NLM35792379 OR NLM35831532 OR NLM35822159 OR NLM35674875 OR NLM35051991 OR NLM34690250 OR NLM34232788 OR NLM35439780 OR NLM34599632 OR NLM35418421 OR NLM35418421 OR NLM34487198 OR NLM35260626 OR NLM35145252 OR NLM34767077 OR NLM35226994 OR NLM35206173 OR NLM34737152 OR NLM34219158 OR NLM34849588 OR NLM34928816 OR NLM34413210 OR NLM34904496 OR NLM33622782 OR NLM33834170 OR NLM33748539 OR NLM34824802 OR NLM34104596 OR NLM33995141 OR NLM33841183 OR NLM34815273 OR NLM34125153 OR NLM33813968 OR NLM34948768 OR NLM35377986 OR NLM35192491 OR NLM34488150 OR NLM34551112 OR NLM33491873 OR NLM34251957 OR NLM34722765 OR NLM34579658 OR NLM33201246 OR NLM33558966 OR NLM33969126 OR NLM34018403 OR NLM34575881 OR NLM34353630 OR NLM34166137 OR NLM34102599 OR NLM34096425 OR NLM34050795 OR NLM33852334 OR NLM33810210 OR NLM33781135 OR NLM33596867 OR NLM33550437 OR NLM32697336 OR NLM33511906 OR NLM33487626 OR NLM33401929 OR NLM33268668 OR NLM33039210 OR NLM32895023 OR NLM32265417 OR NLM32766566 OR NLM32695969 OR NLM32617471 OR NLM32596013 OR NLM33110496 OR NLM32850531 OR NLM32842687 OR NLM32819178 OR NLM32698223 OR NLM33584665 OR NLM33317454 OR NLM33297648 OR NLM33271884 OR NLM33269898 OR NLM33083827 OR NLM33059619 OR NLM33042933 OR NLM32970584 OR NLM32926243 OR NLM32866895 OR NLM32847449 OR NLM32722586 OR NLM32697443 OR NLM32690382 OR NLM32665209 OR NLM31780790 OR NLM31874903 OR NLM32559356 OR NLM32521961 OR NLM32512234 OR NLM32498373 OR NLM32468939 OR NLM32447888 OR NLM32432519 OR NLM32274737 OR NLM32248269 OR NLM32242280 OR NLM32212409 OR NLM32174353 OR NLM32174268 OR NLM32138974 OR NLM32114501 OR NLM32097908 OR NLM32079428 OR NLM32046214 OR NLM31982412 OR NLM31963313 OR NLM31916412 OR NLM31882159 OR NLM31857218 OR NLM31845385 OR NLM31838638 OR NLM31828843 OR NLM31773540 OR NLM31764604 OR NLM31601511 OR NLM31599473 OR NLM31550699 OR NLM31352027 OR NLM31240398 OR NLM31155486 OR NLM31067130 OR NLM30765608 OR NLM30668286 OR NLM31814938 OR NLM31543966 OR NLM30800423 OR NLM30335595 OR NLM32308892 OR NLM31869395 OR NLM31819324 OR NLM31662399 OR NLM31319746 OR NLM31284736 OR NLM31232052 OR NLM31205209 OR NLM31185996 OR NLM31178469 OR NLM31116760 OR NLM31114965 OR NLM31077613 OR NLM31068499 OR NLM30979369 OR NLM30905726 OR NLM30887620 OR NLM30796476 OR NLM30759884 OR NLM30707727 OR NLM30649009 OR NLM30615593 OR NLM30598150 OR NLM30566363 OR NLM30277854 OR NLM30230913 OR NLM29796606 OR NLM31763206 OR NLM31226876 OR NLM31069130 OR NLM29896332 OR NLM30544474 OR NLM30543205 OR NLM30511769 OR NLM30389166 OR NLM30367094 OR NLM29959455 OR NLM29849876 OR NLM29794555 OR NLM29786782 OR NLM29748362 OR NLM29669967 OR NLM29615787 OR NLM29614802 OR NLM29611041 OR NLM29593172 OR NLM29467112 OR NLM29465774 OR NLM29313407 OR NLM29274045 OR NLM29130963 OR NLM29098424 OR NLM29040454 OR NLM29024973 OR NLM28963747 OR NLM28833858 OR NLM28375782 OR NLM29187945 OR NLM28814584 OR NLM28614469 OR NLM29466184 OR NLM29224662 OR NLM29224657 OR NLM29111334 OR NLM29059681 OR NLM28971637 OR NLM28924267 OR NLM28923188 OR NLM28916558 OR NLM28847906 OR NLM28692002 OR NLM28619378 OR NLM28576436 OR NLM28486343 OR NLM28442577 OR NLM28441382 OR NLM28376836 OR NLM28347188 OR NLM28257292 OR NLM28194716 OR NLM28183139 OR NLM28156158 OR NLM28069021 OR NLM27928705 OR NLM27830357 OR NLM27773752 OR NLM27687049 OR NLM27076261 OR NLM26992412 OR NLM26811352 OR NLM28674611 OR NLM28536575 OR NLM28107869 OR NLM27056124 OR NLM26467761 OR NLM28639862 OR NLM28290904 OR NLM28009392 OR NLM27984046 OR NLM27930477 OR NLM27756354 OR NLM27532642 OR NLM27511337 OR NLM27497777 OR NLM27414008 OR NLM27396538 OR NLM27379667 OR NLM27376892 OR NLM27287502 OR NLM27245641 OR NLM27207150 OR NLM27198191 OR NLM27113309 OR NLM27104805 OR NLM27081695 OR NLM27076599 OR NLM27070477 OR NLM26972869 OR NLM26940896 OR NLM26873990 OR NLM26858430 OR NLM26857924 OR NLM26614494 OR NLM26228659 OR NLM24894407 OR NLM27660715 OR NLM27095887 OR NLM26913199 OR NLM26682743 OR NLM26549394 OR NLM26487267 OR NLM26453313 OR NLM26310589 OR NLM26232234 OR NLM26215469 OR NLM26201003 OR NLM26082313 OR NLM26040512 OR NLM25915885 OR NLM25786401 OR NLM25767862 OR NLM25719984 OR NLM25362661 OR NLM25362516 OR NLM25349029 OR NLM25332463 OR NLM25323301 OR NLM25288095 OR NLM24719083 OR NLM26732516 OR NLM25821470 OR NLM24675359 OR NLM24482243 OR NLM25376409 OR NLM25187988 OR NLM25168972 OR NLM25072825 OR NLM25068265 OR NLM25046320 OR NLM25046318 OR NLM25022738 OR NLM24953089 OR NLM24944036 OR NLM24872493 OR NLM24796226 OR NLM24717852 OR NLM24569554 OR NLM24568569 OR NLM24458353 OR NLM24033699 OR NLM24025659 OR NLM23460604 OR NLM24790403 OR NLM24602344 OR NLM27024472 OR NLM24640832 OR NLM24449976 OR NLM24019174 OR NLM23964589 OR NLM23952346 OR NLM23925396 OR NLM23880723 OR NLM23758843 OR NLM23748721 OR NLM23746394 OR NLM23719543 OR NLM23696854 OR NLM23690265 OR NLM23614730 OR NLM23523470 OR NLM23499430 OR NLM23493379 OR NLM23435448 OR NLM23363434 OR NLM23363133 OR NLM23351845 OR NLM23340031 OR NLM23334903 OR NLM23324695 OR NLM23249568 OR NLM23199168 OR NLM22683167 OR NLM22082822 OR NLM23874068 OR NLM23146826 OR NLM23140921 OR NLM22918380 OR NLM22879113 OR NLM22813435 OR NLM22767870 OR NLM22716277 OR NLM22708722 OR NLM22621355 OR NLM22515415 OR NLM22494815 OR NLM22482790 OR NLM22457748 OR NLM22362851 OR NLM22347441 OR NLM22317392 OR NLM22306176 OR NLM22265452 OR NLM22261822 OR NLM22245460 OR NLM22192302 OR NLM22091987 OR NLM22062896 OR NLM21953310 OR NLM21903367 OR NLM21900367 OR NLM21455800 OR NLM22457684 OR NLM21808500 OR NLM22188900 OR NLM22117767 OR NLM22037099 OR NLM21892899 OR NLM21880287 OR NLM21797781 OR NLM21792179 OR NLM21764158 OR NLM21697626 OR NLM21672368 OR NLM21640226 OR NLM21536769 OR NLM21324067 OR NLM21297153 OR NLM21247546 OR NLM21204622 OR NLM21145027 OR NLM21128931 OR NLM21107332 OR NLM20602750 OR NLM21203463 OR NLM21141454 OR NLM21092618 OR NLM20846058 OR NLM20832020 OR NLM20740351 OR NLM20618695 OR NLM20603084 OR NLM20584358 OR NLM20507901 OR NLM20351338 OR NLM20237824 OR NLM20144696 OR NLM20106477 OR NLM20087536 OR NLM19895780 OR NLM19804986 OR NLM19736177 OR NLM19701751 OR NLM19697323 OR NLM19881951 OR NLM19902504 OR NLM19877763 OR NLM19858982 OR NLM19796569 OR NLM19670313 OR NLM19580972 OR NLM19580496 OR NLM19572477 OR NLM19410368 OR NLM19407805 OR NLM19319982 OR NLM19284605 OR NLM19242279 OR NLM19215924 OR NLM19199360 OR NLM19184266 OR NLM19165621 OR NLM18307781 OR NLM17393165 OR NLM18819995 OR NLM19463384 OR NLM19400524 OR NLM19181016 OR NLM19145816 OR NLM19096491 OR NLM19014781 OR NLM19010843 OR NLM18815150 OR NLM18628697 OR NLM18391652 OR NLM18321520 OR NLM18310774 OR NLM18297195 OR NLM18266977 OR NLM18240990 OR NLM18202011 OR NLM17561943 OR NLM17554539 OR NLM19238648 OR NLM18273976 OR NLM18217257 OR NLM17900004 OR NLM17606728 OR NLM17545548 OR NLM17497974 OR NLM17485874 OR NLM17459681 OR NLM17458650 OR NLM17364583 OR NLM17329220 OR NLM17285221 OR NLM17234361 OR NLM17097166 OR NLM16710697 OR NLM16377413 OR NLM16954481 OR NLM16877730 OR NLM16827808 OR NLM16685440 OR NLM16598598 OR NLM16567564 OR NLM16428928 OR NLM16435433 OR NLM16388726 OR NLM16301095 OR NLM16241923 OR NLM16204432 OR NLM16046611 OR NLM15930363 OR NLM15814654 OR NLM15805338 OR NLM15701874 OR NLM15565458 OR NLM15623642 OR NLM15673094 OR NLM15640778 OR NLM15615476 OR NLM15457367 OR NLM15347779 OR NLM15276596 OR NLM15218747 OR NLM15133229 OR NLM15050508 OR NLM15036659 OR NLM15005678 OR NLM14962820 OR NLM15456711 OR NLM14627895 OR NLM12960735 OR NLM12937187 OR NLM12869390 OR NLM12830447 OR NLM12821274 OR NLM12767957 OR NLM12739982 OR NLM12673734 OR NLM12552252 OR NLM12476362 OR NLM12423578 OR NLM12353236 OR NLM12222070 OR NLM12167189 OR NLM12114444 OR NLM12071548 OR NLM11850553 OR NLM11836462 OR NLM21318803 OR NLM14564902 OR NLM11902515 OR NLM11881367 OR NLM11742695 OR NLM11681035 OR NLM11487011 OR NLM11403349 OR NLM11348065 OR NLM10969807 OR NLM10914731 OR NLM10862494 OR NLM10766175 OR NLM10698245 OR NLM10593632 OR NLM10593631 OR NLM10545553 OR NLM10212162 OR NLM12793961 OR NLM9730580 OR NLM9669248 OR NLM9233740 OR NLM9185648 OR NLM9166272 OR NLM9081265 OR NLM8800164 OR NLM8773915 OR NLM8697140 OR NLM8777753 OR NLM8525226 OR NLM8520862 OR NLM7757384 OR NLM7536682 OR NLM7803432 OR NLM7740865 OR NLM8402734 OR NLM8401260 OR NLM8260274 OR NLM8214761 OR NLM8128449 OR NLM8093344 OR NLM7682469 OR NLM1596568 OR NLM1490974 OR NLM1479768 OR NLM1290685 OR NLM1992786 OR NLM1991671 OR NLM1853307 OR NLM1825675 OR NLM1818784 OR NLM10107102 OR NLM2722227 OR NLM3348842 OR NLM2977333 OR NLM2443046 OR NLM3995694 OR NLM3988530 OR NLM3842823 OR NLM6732347 OR NLM6437717 OR NLM6242472 OR NLM6679840 OR NLM6345701 OR NLM6085930 OR NLM6982601 OR NLM7465844)	128
S3	S1 AND S2	364
S2	MH “Personnel Staffing and Scheduling” OR MH “Shift Workers” OR MH “Shiftwork” OR MH “Stress, Occupational” OR MH “Work” OR AB “call schedule” OR TI “call schedule” OR AB “consecutive hours” OR AB “consecutive shifts” OR AB “continuous work” OR AB “day shift” OR TI “day shift” OR AB “duty hour” OR TI “duty hour” OR AB “duty hours” OR TI “duty hours” OR AB “evening shift” OR TI “evening shift” OR AB “evening shifts” OR AB “evening work” OR TI “evening work” OR AB “extended shift” OR TI “extended shift” OR AB “extended shifts” OR TI “extended shifts” OR AB “fixed shift” OR AB “fixed shifts” OR AB “hour shift” OR TI “hour shift” OR AB “hour shifts” OR TI “hour shifts” OR AB “morning shift” OR TI “morning shift” OR AB “morning shifts” OR AB “night call” OR TI “night call” OR AB “night calls” OR TI “night calls” OR AB “night duty” OR TI “night duty” OR AB “night float” OR TI “night float” OR AB “night schedule” OR AB “night schedules” OR AB “night shift” OR TI “night shift” OR AB “night shifts” OR TI “night shifts” OR AB “night work” OR TI “night work” OR AB “night worker” OR AB “night workers” OR TI “night workers” OR AB nightshift OR TI nightshift OR AB nightwork OR TI nightwork OR AB “nocturnal work” OR TI “nocturnal work” OR AB “on-call” OR TI “on-call” OR AB oncall OR AB “overnight shift” OR TI “overnight shift” OR AB “overnight shifts” OR TI “overnight shifts” OR AB (overtime N2 work) OR TI (overtime N2 work) OR AB “overtime work” OR TI “overtime work” OR AB rostering OR TI rostering OR AB “rotating schedules” OR TI “rotating schedules” OR AB “rotating shift” OR TI “rotating shift” OR AB “rotating shifts” OR TI “rotating shifts” OR AB “rotating work” OR TI “rotating work” OR AB “rotation schedule” OR AB “rotation schedules” OR TI “rotation schedules” OR AB “rotation system” OR TI “rotation system” OR AB scheduling OR TI scheduling OR AB “shift cycle” OR AB “shift duration” OR TI “shift duration” OR AB “shift durations” OR TI “shift durations” OR AB (shift* N3 h*) OR TI (shift* N3 h*) OR AB “shift length” OR TI “shift length” OR AB “shift rota” OR AB “shift rotas” OR TI “shift rotas” OR AB “shift rotation” OR TI “shift rotation” OR AB “shift rotations” OR AB “shift schedule” OR TI “shift schedule” OR AB “shift schedules” OR TI “shift schedules” OR AB “shift system” OR TI “shift system” OR AB “shift systems” OR TI “shift systems” OR AB “shift work” OR TI “shift work” OR AB “shift worker” OR TI “shift worker” OR AB “shift workers” OR TI “shift workers” OR AB shiftwork OR TI shiftwork OR AB shiftworker* OR TI shiftworker* OR AB shiftworking OR TI shiftworking OR AB split-shift OR TI split-shift OR AB staffing OR TI staffing OR AB “work hours” OR TI “work hours” OR AB “work schedule” OR TI “work schedule” OR AB “work schedules” OR TI “work schedules” OR AB “work shift” OR TI “work shift” OR AB “work shifts” OR TI “work shifts” OR AB (work* N3 night*) OR TI (work* N3 night*) OR AB (work* N3 shift*) OR TI (work* N3 shift*) OR AB “working hours” OR TI “working hours”	108,869
S1	MH “Atherosclerosis” OR MH “Brachial Artery” OR MH “C-Reactive Protein” OR MH “Carotid Arteries” OR MH “Carotid Intima-Media Thickness” OR MH “Coronary Angiography” OR MH “Coronary Circulation” OR MH “Endothelium” OR MH “Fibrinolysis” OR MH “Hyperemia” OR MH “Hyperhomocysteinemia” OR MH “Interleukins” OR MH “Manometry” OR MH “Nitric Oxide” OR MH “Pulse Wave Velocity” OR MH “Vasodilation” OR AB atherosclerosis OR IT atherosclerosis OR AB “c-reactive protein” OR TI “c-reactive protein” OR AB cIMT OR TI cIMT OR AB “carotid intima-media thickness” OR TI “carotid intima-media thickness” OR AB “carotid ultrasound” OR TI “carotid ultrasound” OR AB “coronary angiography” OR TI “coronary angiography” OR AB crp OR TI crp OR AB endopat OR TI endopat OR AB endothelial OR TI endothelial OR AB endothelium OR TI endothelium OR AB “flow mediated dilation” OR TI “flow mediated dilation” OR AB FMD OR TI FMD OR AB hsCRP OR TI hsCRP OR AB hs-CRP OR TI hs-CRP OR AB hyperemia OR TI hyperemia OR AB IL6 OR TI IL6 OR AB IL-6 OR TI IL-6 OR AB interleukin-6 OR TI interleukin-6 OR AB “intima media thickness” OR TI “intima media thickness” OR AB “nitric oxide” OR TI “nitric oxide” OR AB “NO concentration” OR OR AB PAT OR TI PAT OR AB “peripheral arterial tone” OR TI “peripheral arterial tone” OR AB “peripheral arterial tonometry” OR TI “peripheral arterial tonometry” OR AB plethysmography OR TI plethysmography OR AB “pulse wave” OR TI “pulse wave” OR AB RHI OR AB rh-pat OR AB sphygmomanometer* OR TI sphygmomanometer* OR AB tonometry OR TI tonometry OR AB “vascular function” OR TI “vascular function” OR AB “vascular tone” OR TI “vascular tone” OR AB vasodilation OR TI vasodilation	174,668

## Appendix B. Key Findings

[28] Amir O, Alroy S, Schliamser JE, Asmir I, Shiran A, Flugelman MY, Halon DA, Lewis BS. Brachial artery endothelial function in residents and fellows working night shifts. Am J Cardiol. 2004 Apr 1;93(7):947–9. doi: 10.1016/j.amjcard.2003.12.032. PMID: 15050508. RefID—15 PMID—15050508	
Study Setting	Lady Davis Carmel Medical Center in Israel
Eligible Participants	The study group consisted of 30 healthy physicians (35 ± 4 years of age, range 28 to 45), including 17 internal medicine, five surgery residents, and eight fellows (six cardiology, one gastroenterology, and one hematology).
Study Aims	To examine the effect of night duty, with its inevitable stresses and lifestyle changes, on endothelial function in apparently healthy physicians.
Study Design & Participants	The average duration of the physicians who had worked night shifts at the time of the study was 5 years ± 3 years (range 0.5 to 15). None of the physicians had a history of coronary artery disease, diabetes mellitus, or hypertension. Two physicians were receiving cholesterol-lowering medications (statins), and six were smokers. Investigators used an observational study design comprised of two days (approximately) of observation and data collection. The primary outcome of interest was endothelial function, which was examined twice for each subject: (1) on a regular workday (with no previous or subsequent night shift, defined as baseline measurement of FMD), and (2) after a continuous workday of 24 h, including a night shift. All examinations were performed in the morning (8 to 10 a.m.) under identical conditions in a temperature-controlled room (20 °C to 25 °C). After the shift, physicians completed a questionnaire regarding the shift, including number of hours slept, estimated difficulty of the shift (on a 1 to 10 scale), and level of coffee consumption (number of cups during the shift). Cigarette smoking during the shift was not quantified because smoking is not permitted within the facility. Sleep was documented with a questionnaire.
Intervention/Exposure	Participants completed a night shift (24 h in duration) and sleeping during night shifts was measured post-night shift with a questionnaire.
Comparison(s)	After a 24 h shift (which included night shift work), FMD was measured and compared to a baseline measurement taken on a regular workday.
Outcome Measure(s)	The primary outcome of interest was endothelial function as measured via FMD: (%) = [(D2 − D1)/D1] × 100, where D2 is the reactive hyperemia diameter and D1 is the baseline diameter. In addition, investigators used nitrate-mediated dilation with sub-lingual spray of 400 ug glyceryl trinitrate. Authors reported main findings in Figure 1, showing the impact of night shift exposure on FMD.
Key Finding(s)	Following use of multivariate stepwise regression analyses, as reported in Table 2 of Amir et al., 2004 [28], investigators show that the fewer number of sleeping hours during the shift was associated with greater decrease in FMD post-24 h shift compared to baseline FMD measure (*p* = 0.03). In addition, authors show that the decrease in FMD after the 24 h shift was independently related to a longer history of shift work (*p* = 0.0008).

[47] Garu A, Nitta E, Yoshida Y, Yata E, Tsunematsu A, Araki T, Nagai A, Yano S. Does overnight duty affect vascular endothelial function? BMC Cardiovasc Disord. 2021 Sep 27;21(1):467. doi: 10.1186/s12872-021-02277-y. PMID: 34579658; PMCID: PMC8474775. RefID—167 PMID—34579658	
Study Setting	Clinical research laboratory, Shimane University Hospital, Japan
Eligible Participants	Healthy volunteers over the age of 20 years who had nighttime duty from Shimane University Hospital.
Study Aims	The primary endpoint in this study was the changes in endothelial function after overnight duty. The secondary endpoint was the relationship between endothelial function and fatigue or sleep.
Study Design & Participants	Healthy volunteers were recruited over the age of 20 years who had nighttime duty from Shimane University Hospital. In total, 13 individuals participated in this study with a mean age of 31.6 ± 8.6 years. Investigators used an observational study design and sought to capture a total of six EndoPAT measurements for each subject (three measurements before daytime work and three measurements after nighttime work). Duration of shifts was not reported. All measurements were independently performed in the morning before breakfast. The method of measuring sleep during nighttime work was not reported, yet findings of sleep during nighttime work appear in Table 1 in Garu et al., 2021 [47].
Intervention/Exposure	Exposure of interest was nighttime work: duration of nighttime work was not reported. Sleep during nighttime work was assessed, but the methods of measurement were not reported.
Comparison(s)	After nighttime work, endothelial function was measured (three measures) and compared to a baseline measurement taken before daytime work (three measures).
Outcome Measure(s)	The primary outcome for this study was changes in endothelial function after overnight duty compared to baseline measures, as assessed via the EndoPAT device (outcome reported as Reactive Hyperemia Index RHI).
Key Finding(s)	As reported in Table 1 of the paper, RHI: Before duty 2.12 ± 0.53, after overnight duty 1.97 ± 0.50, *p*-value = 0.21. Sleep hours before duty 6.0 ± 1.2, sleep hours after overnight duty 2.3 ± 1, *p*-value < 0.05.

[48] Tarzia P, Milo M, Di Franco A, Di Monaco A, Cosenza A, Laurito M, Lanza GA, Crea F. Effect of shift work on endothelial function in young cardiology trainees. Eur J Prev Cardiol. 2012 Oct;19(5):908–913. doi: 10.1177/1741826711422765. Epub 2011 Sep 7. PMID: 21900367. RefID—495 PMID—21900367	
Study Setting	Department of Cardiovascular medicine, Catholic University of the Sacred Heart, Rome, Italy.
Eligible Participants	Healthy cardiology trainees affiliated with the university hospital.
Study Aims	This study assessed the acute effect of night work on endothelial function in young medical doctors without any apparent cardiovascular risk factor.
Study Design & Participants	This observational study design involved 20 healthy cardiology trainees without cardiovascular risk factors. These trainees have a history of shift work of 24 ± 12 months and average of 3–4 nights and 1–2 weekend shifts a month. The assessment of the endothelial function was taken at two separate times: one after a working night and another after a restful night. The two sessions were performed in random order. Study supervisors observed the trainees on a regular workday from 8:30 a.m. to 4–6 p.m., while night shifts were observed from 8 p.m. to 8 a.m.
Intervention	The trainee could rest or sleep during shift, when no medical intervention was required, in a dedicated room.
Comparison(s)	After a working night, endothelial function with FMD was captured among trainees. Trainees during a working night in which they obtained rest/sleep were compared to trainees during a restful night.
Outcome Measure(s)	FMD: Subjects rested in a supine position for at least 10 min in a warm, quiet room (22–24 °C) before being tested. A 10 MHz multifrequency linear array probe attached to a high-resolution ultrasound machine was used to acquire images of the right brachial artery. After baseline images of the right brachial artery were obtained for 1 min, a forearm cuff—positioned 1 cm under the antecubital fossa—was inflated to 250 mmHg. The cuff was released 5 min after the inflation to induce forearm reactive hyperemia. Brachial artery diameter was analyzed using automated edge-detection software. Outcomes reported as FMD are seen in Figure 1 and Table 2.
Key Finding(s)	The difference in FMD between WN and RN was not influenced by the number of hours slept during WN. FMD was 8.02 ± 1.4% and 8.56 ± 1.7% after WN and RN, respectively (*p* value 0.025). As seen in Table 1., FMD when trainees had <4 h of sleep during shift was 8.57, while >4 h of sleep during shift was 8.66 (*p*-value = 0.5), as seen in Table 2.

[50] Zheng H, Patel M, Hryniewicz K, Katz SD. Association of extended work shifts, vascular function, and inflammatory markers in internal medicine residents: a randomized crossover trial. JAMA. 2006 Sep 6;296(9):1049–1050. doi: 10.1001/jama.296.9.1049. PMID: 16954481. RefID-595 PMID-16954481	
Study Setting	Clinical research laboratory, Yale University, New Haven, CT
Eligible Participants	Internal Medicine Residents who did not consume caffeine and other unspecified medications
Study Aims	The study aims to test the hypothesis that sleep loss during extended work shifts is associated with evidence of vascular inflammation and dysfunction.
Study Design & Participants	A prospective single-blind, crossover design during an intensive care unit rotation. Participants were assigned at random to two study sessions in random order. One of these sessions was conducted at 1 p.m. after completion of a 30 h extended work shift (from 7 a.m. until 1 p.m. the following day). The other session was conducted at 1 p.m. after completion of a 6 h nonextended work shift (from 7 a.m. until 1 p.m. on the same day). Participants fasted from 7 a.m. to 1 p.m. on the study day.
Intervention	Participants completed a 30 h extended work shift and a 6 h non-extended work shift. After each session, sleep hours from the past night were recorded with a written diary. Flow-mediated dilation of the brachial artery was obtained non-invasively using ultrasonography.
Comparison(s)	FMD measurements taken after the 30 h shift were compared to those taken after the 6 h shift.
Outcome Measure(s)	Sleep duration was self-reported with a written diary. FMD was measured using high-resolution ultrasound imaging.
Key Finding(s)	Sleep duration was found to differ significantly between the extended work group and the non-extended work group, with a median of 0.3 h and 6.5 h, respectively. The *p*-value for this comparison was <0.001. FMD was also found to differ significantly between the extended work group and the non-extended work group, with a median of 3.2% and 7.9%, respectively. The *p*-value for this comparison was also <0.001.

[49] Wehrens SM, Hampton SM, Skene DJ. Heart rate variability and endothelial function after sleep deprivation and recovery sleep among male shift and non-shift workers. Scand J Work Environ Health. 2012 Mar;38(2):171–181. doi: 10.5271/sjweh.3197. Epub 2011 Sep 27. PMID: 21953310. RefID-556 PMID-21953310	
Study Setting	Clinical investigation unit. University of Surrey, United Kingdom
Eligible Participants	Male shift and non-shift workers aged between 25–45 years.
Study Aims	The aim of this study was to investigate the effect of one night of total sleep deprivation, a recovery nap, and recovery sleep on FMD and HRV in controlled laboratory conditions and to assess the responses of experienced shift compared to non-shift workers in the same study.
Study Design & Participants	This experimental study design consisted of eleven experienced male shift workers who had a history of shift work ≥5 years and 14 non-shift workers that were matched for age, body mass index, and cholesterol levels. HRV parameters [e.g., HR variance and low frequency/high frequency]. They spent four days and nights in a clinical investigation unit. Following an adaption night of sleep of around 8 h, the subjects stayed awake for 30.5 h with a 4 h recovery nap.
Intervention	Participants had an adaptation night and baseline night. Subjects were required to stay awake for 30.5 h until their 4 h recovery nap. After this, they had a recovery sleep at habitual bedtime. Subjects were continuously monitored during wake time. All interventions and measurements were scheduled to each subject’s self-selected wake-up time. The subjects were asked to sleep in a semi-recumbent position. Assessments were completed in supine positioning.
Comparison(s)	A baseline FMD was measured from the shift worker group and the non-shift worker group. FMD was next measured after 30.5 h of awake time. FMD was last measured after a 4 h nap following sleep deprivation. FMD can be seen from both comparison groups in Figure 3 in Wehrens et al., 2012 [49].
Outcome Measure(s)	The primary outcome of this study was to compare HRV and endothelial function after sleep deprivation among shift workers. Polysomnography was used to assess wakefulness during the sleep deprivation period. High resolution ultrasound machines were used to access endothelial function of the brachial artery. FMD used 220 frames, ECG triggered images, ultrasound probe, and brachial artery location.
Key Finding(s)	As seen in Figure 3 in Wehrens et al., 2012, there is a trend of lower FMD among shift workers. It is difficult to determine quantitative results from Figure 3 (in Wehrens et al., 2012) displaying the comparison group FMD data. Contradictory statements made from the results section: “The %FMD among both shift and non-shift workers in the morning and afternoon is shown in Figure 3 in Wehrens et al., 2012. There were no significant effects of day, time, group, or interactions on %FMD.”

## Appendix C. Exclusion Tables

1	
RefID	78
PMID	27245641
Citation/Reference	Charles LE, Zhao S, Fekedulegn D, Violanti JM, Andrew ME, Burchfiel CM. Shiftwork and decline in endothelial function among police officers. Am J Ind Med. 2016 Nov;59(11):1001–1008. doi: 10.1002/ajim.22611. Epub 2016 Jun 1. PMID: 27245641; PMCID: PMC5069123.
Exclusion Criteria	Reviewer’s Justification
-Population of interest	
-Interventions of interest	No report of sleep/nap during shift work
-Comparisons of interest	Study lacks the comparison of interest (indicators of endothelial function stratified by sleep/nap vs. no sleep/nap during shift work)
-Outcomes of interest	

2	
RefID	86
PMID	26549394
Citation/Reference	Chou LP, Li CY, Hu SC. Work-Related Psychosocial Hazards and Arteriosclerosis. Int Heart J. 2015;56(6):644–650. doi: 10.1536/ihj.15-143. Epub 2015 Nov 9. PMID: 26549394.
Exclusion Criteria	Reviewer’s Justification
-Population of interest	
-Interventions of interest	No report of sleep/nap during shift work
-Comparisons of interest	Study lacks the comparison of interest (indicators of endothelial function stratified by sleep/nap vs. no sleep/nap during shift work)
-Outcomes of interest	

3	
RefID	122
PMID	35418421
Citation/Reference	Draaijer M, Scheuermaier K, Lalla-Edward ST, Fischer AE, Grobbee DE, Venter F, Vos A. Influence of shift work on cardiovascular disease risk in Southern African long-distance truck drivers: a cross-sectional study. BMJ Open. 2022 Apr 13;12(4):e050645. doi: 10.1136/bmjopen-2021-050645. PMID: 35418421; PMCID: PMC9013993.
Exclusion Criteria	Reviewer’s Justification
-Population of interest	
-Interventions of interest	No report of sleep/nap during shift work
-Comparisons of interest	Study lacks the comparison of interest (indicators of endothelial function stratified by sleep/nap vs. no sleep/nap during shift work)
-Outcomes of interest	

4	
RefID	164
PMID	12423578
Citation/Reference	García-Fernández R, García Pérez-Velasco J, Milián AC, Peix González A, García-Barreto D. Disfunción endotelial en cardiólogos tras una guardia médica [Endothelial dysfunction in cardiologists after 24 h on call]. Rev Esp Cardiol. 2002 Nov;55(11):1202–1204. Spanish. doi: 10.1016/s0300-8932(02)76784-5. PMID: 12423578.
Exclusion Criteria	Reviewer’s Justification
-Population of interest	
-Interventions of interest	No report of sleep/nap during shift work
-Comparisons of interest	Study lacks the comparison of interest (indicators of endothelial function stratified by sleep/nap vs. no sleep/nap during shift work)
-Outcomes of interest	

5	
RefID	238
PMID	21697626
Citation/Reference	Kamata N, Tanaka K, Morita S, Tagaya H, Kawashima M, Shichiri M, Miyaoka H. Relationship between autonomic nervous system activity during sleep and fasting glucose in Japanese workers. Ind Health. 2011;49(4):427–33. doi: 10.2486/indhealth.ms1257. Epub 2011 Jun 21. PMID: 21697626.
Exclusion Criteria	Reviewer’s Justification
-Population of interest	
-Interventions of interest	No report of sleep/nap during shift work
-Comparisons of interest	Study lacks the comparison of interest (indicators of endothelial function stratified by sleep/nap vs. no sleep/nap during shift work)
-Outcomes of interest	No direct measure of endothelial function was present in the study.

6	
RefID	244
PMID	23324695
Citation/Reference	Kantermann T, Duboutay F, Haubruge D, Kerkhofs M, Schmidt-Trucksäss A, Skene DJ. Atherosclerotic risk and social jetlag in rotating shift-workers: first evidence from a pilot study. Work. 2013 Jan 1;46(3):273–282. doi: 10.3233/WOR-121531. PMID: 23324695.
Exclusion Criteria	Reviewer’s Justification
-Population of interest	
-Interventions of interest	It is unlikely that study participants slept during night shifts, and sleep duration was not measured.
-Comparisons of interest	
-Outcomes of interest	No direct measure of endothelial function.

7	
RefID	258
PMID	21903367
Citation/Reference	Kim W, Park CS, Yu TK, Park HH, Cho EK, Kang WY, Hwang SH, Lee ES, Kim W. The preventive effects of dark chocolate on impaired endothelial function in medical personnel working sequential night shifts. Nutr Metab Cardiovasc Dis. 2012 Feb;22(2):e3–e4. doi: 10.1016/j.numecd.2011.04.001. Epub 2011 Sep 7. PMID: 21903367.
Exclusion Criteria	Reviewer’s Justification
-Population of interest	
-Interventions of interest	No report of sleep/nap during shift work
-Comparisons of interest	Study lacks the comparison of interest (indicators of endothelial function stratified by sleep/nap vs. no sleep/nap during shift work)
-Outcomes of interest	

8	
RefID	259
PMID	21764158
Citation/Reference	Kim W, Park HH, Park CS, Cho EK, Kang WY, Lee ES, Kim W. Impaired endothelial function in medical personnel working sequential night shifts. Int J Cardiol. 2011 Sep 15;151(3):377–378. doi: 10.1016/j.ijcard.2011.06.109. Epub 2011 Jul 20. PMID: 21764158.
Exclusion Criteria	Reviewer’s Justification
-Population of interest	
-Interventions of interest	No report of sleep/nap during shift work
-Comparisons of interest	Study lacks the comparison of interest (indicators of endothelial function stratified by sleep/nap vs. no sleep/nap during shift work)
-Outcomes of interest	

9	
RefID	306
PMID	31963313
Citation/Reference	Lunde LK, Skare Ø, Mamen A, Sirnes PA, Aass HCD, Øvstebø R, Goffeng E, Matre D, Nielsen P, Heglum HSA, Hammer SE, Skogstad M. Cardiovascular Health Effects of Shift Work with Long Working Hours and Night Shifts: Study Protocol for a Three-Year Prospective Follow-Up Study on Industrial Workers. Int J Environ Res Public Health. 2020 Jan 16;17(2):589. doi: 10.3390/ijerph17020589. PMID: 31963313; PMCID: PMC7014249.
Exclusion Criteria	Reviewer’s Justification
-Population of interest	Protocol paper, no data presented
-Interventions of interest	
-Comparisons of interest	
-Outcomes of interest	

10	
RefID	322
PMID	35206173
Citation/Reference	Matre D, Sirnes PA, Goffeng E, Skare Ø, Skogstad M. Sleep Duration, Number of Awakenings and Arterial Stiffness in Industrial Shift Workers: A Five-Week Follow-Up Study. Int J Environ Res Public Health. 2022 Feb 10;19(4):1964. doi: 10.3390/ijerph19041964. PMID: 35206173; PMCID: PMC8872215.
Exclusion Criteria	Reviewer’s Justification
-Population of interest	
-Interventions of interest	No report of sleep/nap during shift work
-Comparisons of interest	Study lacks the comparison of interest (indicators of endothelial function stratified by sleep/nap vs. no sleep/nap during shift work)
-Outcomes of interest	Primary outcome was arterial stiffness by measuring the pulse wave velocity

11	
RefID	442
PMID	21107332
Citation/Reference	Shimada K, Fukuda S, Maeda K, Kawasaki T, Kono Y, Jissho S, Taguchi H, Yoshiyama M, Yoshikawa J. Aromatherapy alleviates endothelial dysfunction of medical staff after night-shift work: preliminary observations. Hypertens Res. 2011 Feb;34(2):264–267. doi: 10.1038/hr.2010.228. Epub 2010 Nov 25. PMID: 21107332.
Exclusion Criteria	Reviewer’s Justification
-Population of interest	Medical staff
-Interventions of interest	Subjects stated their mean sleep time during shift; however, this was not analyzed as a variable. The main intervention was a 30 min rest period after a completed night shift with aromatherapy via inhalation.
-Comparisons of interest	
-Outcomes of interest	

12	
RefID	478
PMID	31780790
Citation/Reference	Sugiura T, Dohi Y, Takagi Y, Yoshikane N, Ito M, Suzuki K, Nagami T, Iwase M, Seo Y, Ohte N. Impacts of lifestyle behavior and shift work on visceral fat accumulation and the presence of atherosclerosis in middle-aged male workers. Hypertens Res. 2020 Mar;43(3):235–245. doi: 10.1038/s41440-019-0362-z. Epub 2019 Nov 28. PMID: 31780790.
Exclusion Criteria	Reviewer’s Justification
-Population of interest	10,073 male shift workers
-Interventions of interest	No report of sleep/nap during shift work
-Comparisons of interest	Study lacks the comparison of interest (indicators of endothelial function stratified by sleep/nap vs. no sleep/nap during shift work)
-Outcomes of interest	

13	
RefID	539
PMID	25349029
Citation/Reference	Wang A, Arah OA, Kauhanen J, Krause N. Work schedules and 11-year progression of carotid atherosclerosis in middle-aged Finnish men. Am J Ind Med. 2015 Jan;58(1):1–13. doi: 10.1002/ajim.22388. Epub 2014 Oct 27. PMID: 25349029.
Exclusion Criteria	Reviewer’s Justification
-Population of interest	Finnish shift workers
-Interventions of interest	No report of sleep/nap during shift work
-Comparisons of interest	Study lacks the comparison of interest (indicators of endothelial function stratified by sleep/nap vs. no sleep/nap during shift work)
-Outcomes of interest	

14	
RefID	719
PMID	N/A
Citation/Reference	Chou, L., et al. “Job Stress, Mental Health, Burnout and Arterial Stiffness: A Cross-Sectional Study among Taiwanese Medical Professionals.” *Atherosclerosis*, vol. 241, no. 1, July 2015, p. E135., https://doi.org/10.1016/j.atherosclerosis.2015.04.468.
Exclusion Criteria	Reviewer’s Justification
-Population of interest	Conference abstract/abstract only
-Interventions of interest	
-Comparisons of interest	
-Outcomes of interest	

15	
RefID	720
PMID	N/A
Citation/Reference	Choudhary, Arbind Kumar, et al. “Sleep restriction and its influence on blood pressure.” *Artery Research* 19 (2017): 42–48.
Exclusion Criteria	Reviewer’s Justification
-Population of interest	
-Interventions of interest	No report of sleep/nap during shift work
-Comparisons of interest	Study lacks the comparison of interest (indicators of endothelial function stratified by sleep/nap vs. no sleep/nap during shift work)
-Outcomes of interest	

16	
RefID	477
PMID	21247546
Citation/Reference	Suessenbacher A, Potocnik M, Dörler J, Fluckinger G, Wanitschek M, Pachinger O, Frick M, Alber HF. Comparison of peripheral endothelial function in shift versus nonshift workers. Am J Cardiol. 2011 Mar 15;107(6):945–8. doi: 10.1016/j.amjcard.2010.10.077. Epub 2011 Jan 19. PMID: 21247546.
Exclusion Criteria	Reviewer’s Justification
-Population of interest	Duplicate record for an exclusion (see Appendix C, RefID 1221 in Appendix C).
-Interventions of interest	
-Comparisons of Interest	
-Outcomes of interest	

17	
RefID	865
PMID	N/A
Citation/Reference	Jo Eun-kiung et al. “The Effect of Cocoa on Impaired Flow-Mediated Dilation in Working Night Shifts.” Korean Society of Lipid and Arteriosclerosis Fall Conference Proceedings. 2009 (2009): 132–132.
Exclusion Criteria	Reviewer’s Justification
-Population of interest	Conference abstract/abstract only
-Interventions of interest	
-Comparisons of interest	
-Outcomes of interest	

18	
RefID	898
PMID	N/A
Citation/Reference	Taleen Khalaf, Pratik Dalal, Divyashree Varma, ShukoLee, Robert Chilton, ReneOliveros,Arterialstiffnessdetermined fromambulatory blood pressure monitoring,Journal oftheAmerican Society of Hypertension,Volume 10, Issue 4, Supplement,2016,Page e29,ISSN19331711, https://doi.org/10.1016/j.jash.2016.03.068. (https://www.sciencedirect.com/science/article/pii/S1933171116300717) Accessed on 1 June 2023.
Exclusion Criteria	Reviewer’s Justification
-Population of interest	Conference abstract/abstract only
-Interventions of interest	
-Comparisons of interest	
-Outcomes of interest	

19	
RefID	907
PMID	N/A
Citation/Reference	Jo Eun-kiung, et al. “The Effect of Cocoa on Impaired Flow-Mediated Dilation in Working Night Shifts.” Journal of the Korean Society of Lipid and Arteriosclerosis Fall Conference. 2009 (2009): 132–132.
Exclusion Criteria	Reviewer’s Justification
-Population of interest	Duplicate record for an exclusion (see Appendix C, RefID 865 in Appendix C).
-Interventions of interest	
-Comparisons of interest	
-Outcomes of interest	

20	
RefID	908
PMID	21903367
Citation/Reference	Kim W, Park CS, Yu TK, Park HH, Cho EK, Kang WY, Hwang SH, Lee ES, Kim W. The preventive effects of dark chocolate on impaired endothelial function in medical personnel working sequential night shifts. Nutr Metab Cardiovasc Dis. 2012 Feb;22(2):e3–e4. doi: 10.1016/j.numecd.2011.04.001. Epub 2011 Sep 7. PMID: 21903367.
Exclusion Criteria	Reviewer’s Justification
-Population of interest	
-Interventions of interest	No report of sleep/nap during shift work
-Comparisons of interest	Study lacks the comparison of interest (indicators of endothelial function stratified by sleep/nap vs. no sleep/nap during shift work)
-Outcomes of interest	

21	
RefID	909
PMID	NA
Citation/Reference	W Kim, CS Park, H Park, W Kim, K-S Kim, Abstract: P348 IMPAIRED ENDOTHELIAL FUNCTION INNURSESWORKINGNIGHTSHIFTS, Atherosclerosis Supplement Volume 10, Issue 2, 2009, Page e657,ISSN 1567-5688, https://doi.org/10.1016/S1567-5688(09)70643-9. (https://www.sciencedirect.com/science/article/pii/S1567568809706439) Accessed on 1 June 2023.
Exclusion Criteria	Reviewer’s Justification
-Population of interest	Conference abstract/abstract only (possibly related to RefID259)
-Interventions of interest	
-Comparisons of interest	
-Outcomes of interest	

22	
RefID	1066
PMID	N/A
Citation/Reference	Özbay, S., Özyılmaz, İ., Uysal, A., Hamidi, M., & Serdar, O. A. (2012). Gece Şartlarında Hastane Ortamında Çalışma Arteriyel Sertlik için Risk Faktörüdür. *Medical Bulletin of Haseki/Haseki Tip Bulteni*, *50*(3).
Exclusion Criteria	Reviewer’s Justification
-Population of interest	Article not in English
-Interventions of interest	
-Comparisons of interest	
-Outcomes of interest	

23	
RefID	1184
PMID	N/A
Citation/Reference	Shurkevich, N., Vetoshkin, A., Gapon, L., Simonyan, A., & Dyachkov, S. (2021). Arterial stiffness as a risk factor for heart failure formation in rotational shift workers in the arctic region. *Atherosclerosis*, *331*, e196.
Exclusion Criteria	Reviewer’s Justification
-Population of interest	Conference abstract/abstract only
-Interventions of interest	
-Comparisons of interest	
-Outcomes of interest	

24	
RefID	1185
PMID	N/A
Citation/Reference	Shurkevich, N., Vetoshkin, A., Gapon, L., Simonyan, A., & Kuznetsov, V. (2020). Risk prediction for subclinical carotid atherosclerosis in rotational shift workers in the arctic. *European Heart Journal*, *41*(Supplement_2), ehaa946-2711.
Exclusion Criteria	Reviewer’s Justification
-Population of interest	Journal Abstract: Study lacks the comparison of interest (indicators of endothelial function stratified by sleep/nap vs. no sleep/nap during shift work)
-Interventions of interest	
-Comparisons of interest	
-Outcomes of interest	

25	
RefID	1194 (Duplicate of RecordID 556 in Appendix B)
PMID	21953310
Citation/Reference	Wehrens SM, Hampton SM, Skene DJ. Heart rate variability and endothelial function after sleep deprivation and recovery sleep among male shift and non-shift workers. Scand J Work Environ Health. 2012 Mar;38(2):171–181. doi: 10.5271/sjweh.3197. Epub 2011 Sep 27. PMID: 21953310.
Exclusion Criteria	Reviewer’s Justification
-Population of interest	Duplicate record for an inclusion (see Appendix B, RefID 556).
-Interventions of interest	
-Comparisons of interest	
-Outcomes of interest	

26	
RefID	1219
PMID	NA
Citation/Reference	Stuchlik, Patrick, et al. “Sleep Duration and Subclinical Measures of Atherosclerosis in a Bi-racial Cohort: The Bogalusa Heart Study.” *Circulation* 136.suppl_1 (2017): A19286-A19286.
Exclusion Criteria	Reviewer’s Justification
-Population of interest	Conference abstract/abstract only
-Interventions of interest	
-Comparisons of interest	
-Outcomes of interest	

27	
RefID	1221
PMID	21247546
Citation/Reference	Suessenbacher A, Potocnik M, Dörler J, Fluckinger G, Wanitschek M, Pachinger O, Frick M, Alber HF. Comparison of peripheral endothelial function in shift versus nonshift workers. Am J Cardiol. 2011 Mar 15;107(6):945–948. doi: 10.1016/j.amjcard.2010.10.077. Epub 2011 Jan 19. PMID: 21247546.
Exclusion Criteria	Reviewer’s Justification
-Population of interest	
-Interventions of interest	No report of sleep/nap during shift work
-Comparisons of interest	Study lacks the comparison of interest (indicators of endothelial function stratified by sleep/nap vs. no sleep/nap during shift work)
-Outcomes of interest	

28	
RefID	1222
PMID	31780790
Citation/Reference	Sugiura T, Dohi Y, Takagi Y, Yoshikane N, Ito M, Suzuki K, Nagami T, Iwase M, Seo Y, Ohte N. Impacts of lifestyle behavior and shift work on visceral fat accumulation and the presence of atherosclerosis in middle-aged male workers. Hypertens Res. 2020 Mar;43(3):235–245. doi: 10.1038/s41440-019-0362-z. Epub 2019 Nov 28. PMID: 31780790.
Exclusion Criteria	Reviewer’s Justification
-Population of interest	Duplicate of RefID478 in Appendix C
-Interventions of interest	
-Comparisons of interest	
-Outcomes of interest	

29	
RefID	1239
PMID	21900367
Citation/Reference	Tarzia P, Milo M, Di Franco A, Di Monaco A, Cosenza A, Laurito M, Lanza GA, Crea F. Effect of shift work on endothelial function in young cardiology trainees. Eur J Prev Cardiol. 2012 Oct;19(5):908–13. doi: 10.1177/1741826711422765. Epub 2011 Sep 7. PMID: 21900367.
Exclusion Criteria	Reviewer’s Justification
-Population of interest	Duplicate record for an inclusion (see Appendix B, RefID 495).
-Interventions of interest	
-Comparisons of interest	
-Outcomes of interest	

30	
RefID	1344
PMID	N/A
Citation/Reference	Yuyan, Guo, et al. “Chronic Sleep Deprivation Caused By Night Shift Work And Arterial Stiffness Among Nurses In Beijing.” *C64. UPPER AIRWAY AND RESPIRATORY CONTROL DURING SLEEP AND NON-PULMONARY SLEEP DISORDERS*. American Thoracic Society, 2014. A5052-A5052.
Exclusion Criteria	Reviewer’s Justification
-Population of interest	Conference abstract/abstract only
-Interventions of interest	
-Comparisons of interest	
-Outcomes of interest	

31	
RefID	1661
PMID	24038303
Citation/Reference	Ma CC, Burchfiel CM, Charles LE, Dorn JM, Andrew ME, Gu JK, Joseph PN, Fekedulegn D, Slaven JE, Hartley TA, Mnatsakanova A, Violanti JM. Associations of objectively measured and self-reported sleep duration with carotid artery intima media thickness among police officers. Am J Ind Med. 2013 Nov;56(11):1341–1351. doi: 10.1002/ajim.22236. Epub 2013 Aug 22. PMID: 24038303; PMCID: PMC4502427.
Exclusion Criteria	Reviewer’s Justification
-Population of interest	
-Interventions of interest	No report of sleep/nap during shift work
-Comparisons of interest	Study lacks the comparison of interest (indicators of endothelial function stratified by sleep/nap vs. no sleep/nap during shift work)
-Outcomes of interest	

32	
RefID	Bibliography search from record 495
PMID	19215924
Citation/Reference	Puttonen, S., Kivimäki, M., Elovainio, M., Pulkki-Råback, L., Hintsanen, M., Vahtera, J., Telama, R., Juonala, M., Viikari, J. S., Raitakari, O. T., & Keltikangas-Järvinen, L. (2009). Shift work in young adults and carotid artery intima-media thickness: The Cardiovascular Risk in Young Finns study. *Atherosclerosis*, *205*(2), 608–613. https://doi.org/10.1016/j.atherosclerosis.2009.01.016
Exclusion Criteria	Reviewer’s Justification
-Population of interest	
-Interventions of interest	No report of sleep/nap during shift work
-Comparisons of interest	Study lacks the comparison of interest (indicators of endothelial function stratified by sleep/nap vs. no sleep/nap during shift work)
-Outcomes of interest	

33	
RefID	Bibliography search from record 167
PMID	15119699
Citation/Reference	Takase B, Akima T, Uehata A, Ohsuzu F, Kurita A. Effect of chronic stress and sleep deprivation on both flow-mediated dilation in the brachial artery and the intracellular magnesium level in humans. Clin Cardiol. 2004 Apr;27(4):223–227. doi: 10.1002/clc.4960270411. PMID: 15119699; PMCID: PMC6654639.
Exclusion Criteria	Reviewer’s Justification
-Population of interest	Male college students
-Interventions of interest	No report of sleep/nap during shift work
-Comparisons of interest	Study lacks the comparison of interest (indicators of endothelial function stratified by sleep/nap vs. no sleep/nap during shift work)
-Outcomes of interest	

34	
RefID	Bibliography search from record 1661
PMID	17289054
Citation/Reference	Wolff B, Völzke H, Schwahn C, Robinson D, Kessler C, John U. Relation of self-reported sleep duration with carotid intima-media thickness in a general population sample. Atherosclerosis. 2008 Feb;196(2):727–732. doi: 10.1016/j.atherosclerosis.2006.12.023. Epub 2007 Feb 6. PMID: 17289054.
Exclusion Criteria	Reviewer’s Justification
-Population of interest	
-Interventions of interest	No report of sleep/nap during shift work
-Comparisons of interest	Study lacks the comparison of interest (indicators of endothelial function stratified by sleep/nap vs. no sleep/nap during shift work)
-Outcomes of interest	

35	
RefID	Bibliography search from record 1661
PMID	22935396
Citation/Reference	Sands MR, Lauderdale DS, Liu K, Knutson KL, Matthews KA, Eaton CB, Linkletter CD, Loucks EB. Short sleep duration is associated with carotid intima-media thickness among men in the Coronary Artery Risk Development in Young Adults (CARDIA) Study. Stroke. 2012 Nov;43(11):2858–2864. doi: 10.1161/STROKEAHA.112.660332. Epub 2012 Aug 30. PMID: 22935396; PMCID: PMC3479367.
Exclusion Criteria	Reviewer’s Justification
-Population of interest	Not shift workers
-Interventions of interest	
-Comparisons of interest	
-Outcomes of interest	

## Appendix D. PRISMA ScR Checklist

**Section**	**Item**	**Location**
Title	Identify the report as a scoping review	In title
Abstract: Structured summary	Provide a structured summary that includes (as applicable) background, objectives, eligibility criteria, sources of evidence, charting methods, results, and conclusions that relate to the review questions and objectives.	Abstract included
Introduction: Rationale	Describe the rationale for the review in the context of what is already known. Explain why the review questions/objectives lend themselves to a scoping review approach.	Included in introduction Section 1
Introduction: Objectives	Provide an explicit statement of the questions and objectives being addressed with reference to their key elements (e.g., population or participants, concepts, and context) or other relevant key elements used to conceptualize the review questions and/or objectives.	Last sentence of introduction section and first paragraph of the methods section
Methods: Protocol and registration	Indicate whether a review protocol exists; state if and where it can be accessed (e.g., a Web address); if available, provide registration information, including the registration number.	Last sentence in methods section, sub-section “Screening methodology”
Methods: Eligibility criteria	Specify characteristics of the sources of evidence used as eligibility criteria (e.g., years considered, language, and publication status) and provide a rationale.	First two paragraphs of methods section
Methods: Information sources	Describe all information sources in the search (e.g., databases with dates of coverage and contact with authors to identify additional sources), as well as the date the most recent search was executed.	First two paragraphs of methods section
Methods: Search	Present the full electronic search strategy for at least one database, including any limits used, such that it could be repeated.	See Appendix A for details for each database searched
Methods: Selection of sources of evidence	State the process for selecting sources of evidence (i.e., screening and eligibility) included in the scoping review.	See third paragraph of methods section, sub-section “Screening methodology”
Methods: Data charting process	Describe the methods of charting data from the included sources of evidence (e.g., calibrated forms or forms that have been tested by the team before their use, and whether data charting was performed independently or in duplicate) and any processes for obtaining and confirming the data from investigators.	See the methods section, sub-section “Analysis”
Methods: Data items	List and define all variables for which data were sought and any assumptions and simplifications made.	See methods section, sub-sections “population of interest, intervention/exposure of interest, comparison of interest, outcome of interest” and Appendix B, where details of each reviewed article was abstracted into tables
Methods: Critical appraisal of individual sources of evidence	If completed, provide a rationale for conducting a critical appraisal of included sources of evidence; describe the methods used and how this information was used in any data synthesis (if appropriate).	See the methods section, sub-section “Analysis”
Methods: Summary measures	Not applicable for scoping reviews.	Not applicable
Methods: Synthesis of results	Describe the methods of handling and summarizing the data that were charted.	See the methods section, sub-section “Analysis”, and see Appendix B
Methods: Risk of bias across studies	Not applicable for scoping reviews.	Not applicable
Methods: Additional analyses	Not applicable for scoping reviews.	Not applicable
Results: Selection of sources of evidence	Give numbers of sources of evidence screened, assessed for eligibility, and included in the review, with reasons for exclusions at each stage, ideally using a flow diagram.	See first paragraph of Results section and Appendix A
Results: Characteristics of sources of evidence	For each source of evidence, present characteristics for which data were charted and provide the citations.	See Appendix B
Results: Critical appraisal within sources of evidence	If completed, present data on critical appraisal of included sources of evidence.	See results section, paragraphs 2 to 8
Results: Results of individual sources of evidence	For each included source of evidence, present the relevant data that were charted that relate to the review questions and objectives.	See Appendix B and paragraphs 2 through 8 of the results section
Results: Synthesis of results	Summarize and/or present the charting results as they relate to the review questions and objectives.	See Appendix B and paragraphs 2 through 8 of the results section
Results: Risk of bias across studies	Not applicable for scoping reviews.	Not applicable
Results: Additional analyses	Not applicable for scoping reviews.	Not applicable
Discussion: Summary of evidence	Summarize the main results (including an overview of concepts, themes, and types of evidence available), link to the review questions and objectives, and consider the relevance to key groups.	First two paragraphs of discussion section
Discussion: Limitations	Discuss the limitations of the scoping review process.	See limitations section
Discussion: Conclusions	Provide a general interpretation of the results with respect to the review questions and objectives, as well as potential implications and/or next steps.	See first two paragraphs of discussion section and the conclusion section
Funding:	Describe sources of funding for the included sources of evidence, as well as sources of funding for the scoping review. Describe the role of the funders of the scoping review.	See acknowledgement section
